# Differential regulation of local mRNA dynamics and translation following long-term potentiation and depression

**DOI:** 10.1073/pnas.2017578118

**Published:** 2021-03-26

**Authors:** Paul G. Donlin-Asp, Claudio Polisseni, Robin Klimek, Alexander Heckel, Erin M. Schuman

**Affiliations:** ^a^Department of Synaptic Plasticity, Max Planck Institute for Brain Research, 60438 Frankfurt, Germany;; ^b^Institute for Organic Chemistry and Chemical Biology, Goethe-University Frankfurt, 60438 Frankfurt, Germany

**Keywords:** mRNA, local protein synthesis, mRNA beacons, mRNA localization, neuron protein synthesis

## Abstract

Decades of work have demonstrated that messenger RNAs (mRNAs) are localized and translated within neuronal dendrites and axons to provide proteins for remodeling and maintaining growth cones or synapses. It remains unknown, however, whether specific forms of plasticity differentially regulate the dynamics and translation of individual mRNA species. To address this, we targeted three individual synaptically localized mRNAs, *CamkIIa*, *β-actin*, *Psd95,* and used molecular beacons to track endogenous mRNA movements. We used reporters and CRISPR/Cas9 gene editing to track mRNA translation in cultured neurons. We found alterations in mRNA dynamic properties occurred during two forms of synaptic plasticity, long-term potentiation (cLTP) and depression (mGluR-LTD). Changes in mRNA dynamics following either form of plasticity resulted in an enrichment of mRNA in the vicinity of dendritic spines. Both the reporters and tagging of endogenous proteins revealed the transcript-specific stimulation of protein synthesis following cLTP or mGluR-LTD. As such, the plasticity-induced enrichment of mRNA near synapses could be uncoupled from its translational status. The enrichment of mRNA in the proximity of spines allows for localized signaling pathways to decode plasticity milieus and stimulate a specific translational profile, resulting in a customized remodeling of the synaptic proteome.

## Significance

Local protein synthesis is important for neuronal function and synaptic plasticity. Thousands of mRNAs are found in axons and dendrites, and it is believed that regulating their dynamic transport and distribution is a key determinant of where and when proteins are made. In this work we quantitatively assessed the dynamic transport of three synaptically localized mRNAs in live cultured neurons without exogenous stimulation and following induction of two distinct forms of synaptic plasticity. Coupling observations of mRNA dynamics with live imaging of endogenous protein synthesis dynamics, we found that alterations in mRNA movements occur independently of their translational state, indicating a multistep mechanism of capture and decoding of an mRNA to determine when translation occurs.

Synaptic plasticity requires the rapid and robust remodeling of the proteome (1). Both the strengthening (long-term potentiation, LTP) and the weakening of synaptic connections (long-term depression, LTD) requires proteome remodeling (2). Neurons use diverse mechanisms to achieve this regulation, including the posttranslational modifications of proteins (2), transcriptional changes (3), and translational changes (4). Indeed, neurons can rapidly regulate and control synaptic proteomes by localizing and translating messenger RNAs (mRNAs) in axons and dendrites (5–12). A number of key synaptic proteins are encoded by translationally regulated mRNAs, including ARC (13–15), fragile X mental retardation protein (FMRP) (16), postsynaptic density 95 (PSD-95) (16, 17), and CAMK2a (18). Given the capacity for protein synthesis in distal compartments, a fundamental question is how dendritically and axonally localized mRNAs become recruited near synapses and then translationally regulated locally during plasticity.

Current evidence suggests that single mRNAs, bound by RNA-binding proteins (RBPs), interact with the cytoskeleton for long-distance transport from the cell body to the dendrites and axons (19, 20). Both localization (19) and translational regulatory elements (21) are present in the 5′ and 3′ untranslated regions (UTRs) of mRNAs. Trans-acting factors, including RBPs and microRNAs (miRNAs), interact with UTR elements to regulate mRNA localization and translation; these interactions are also regulated by plasticity. mRNAs are believed to be transported in a translationally quiescent state, likely only engaged in translation near synapses (22, 23). The dynamic and bidirectional (22, 24–26) scanning behavior of mRNAs in dendrites allows, in principle, for the capture and translation of mRNAs as needed for proteome maintenance and remodeling (22, 23). Our understanding of these processes, however, is largely derived from live imaging experiments for a limited number of individual candidate mRNAs including *β-actin* (22, 25) and *Arc* (27); the relationship between the sequestration/capture of RNAs and their translation during plasticity is not well understood.

To address this, we used molecular beacons to track and quantify the dynamics of three endogenous mRNAs under basal conditions and after plasticity. We found that induction of either chemically induced LTP (cLTP) or metabotropic glutamate receptor LTD (mGluR-LTD) resulted in a widespread attenuation of mRNA motility and led to an enrichment of mRNA near dendritic spines. These altered mRNA dynamics and availability near synapses was accompanied for some, but not all, mRNAs by enhanced translation of either a reporter or a CRISPR/Cas9-tagged endogenous protein. This dissociation allows for the enrichment of mRNAs near spines where localized signaling pathways can control which specific sets of transcripts are translated.

## Results

### Tracking Endogenous mRNA Dynamics in Live Neurons.

**Fig. 1.**
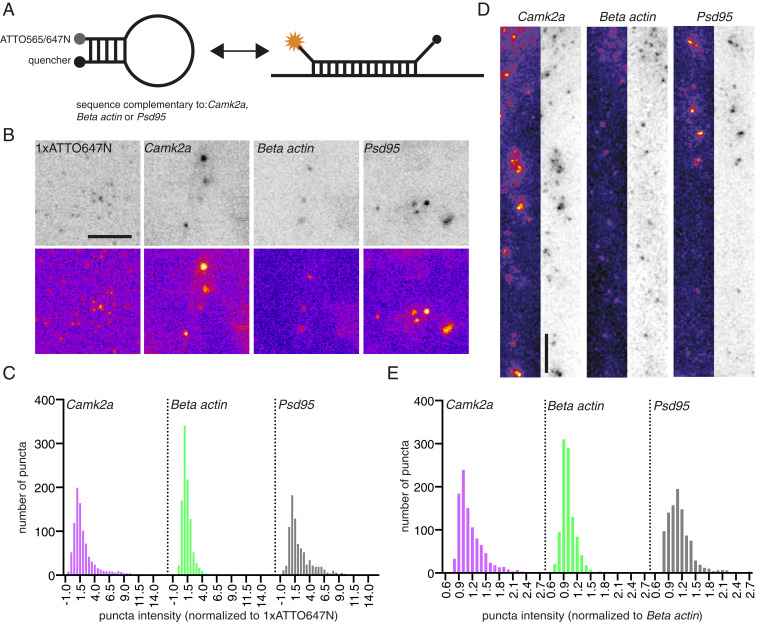
Dendritic mRNAs exist within varied copy number states. (*A*) Scheme of the molecular beacon design used in this study. A 5-nt stem, with a reporter fluorophore on one side and a quencher dye on the other side, were linked with a 26-nt complementary sequence to *Camk2a*, *β-actin*, or *Psd95* mRNAs. When not hybridized to a target mRNA, the stem holds the reporter and quencher in proximity, preventing fluorescence. Upon binding to a target mRNA, the stem loop opens, and fluorescence can be detected in a reversible manner. (*B*) Example images of Gatta quant standard and beacon-labeled neurons shows a heterogenous distribution of particle intensities in live neurons. (Scale bar, 5 μm.) Data shown acquired at 5% laser power. (*C*) Quantification of the distribution of beacon intensity relative to single fluor labeled ATTO647 standard. Atto647N *Camk2a* (19 cells, 921 mRNA granules), Atto647N *β-actin* (21 cells, 1,003 mRNA granules), Atto647N *Psd95* (18 cells, 829 mRNA granules). (*D*) Example smFISH images of dendritically localized *β-actin*, *Camk2a*, and *Psd95*. (Scale bar, 5 μm.) (*E*) Quantification of the distribution of smFISH intensity. *Camk2a* (15 cells, 1,005 mRNA granules), *β-actin* (15 cells, 1,005 mRNA granules), *Psd95* (15 cells, 1,005 mRNA granules).

To assess endogenous mRNA dynamics, we focused on *Camk2a*, *β-actin*, and *Psd95* as they are abundant in neuronal dendrites (28) and are translationally regulated by plasticity (18, 22, 29, 30). To track these mRNAs, we employed molecular beacons (Fig. 1*A*) (31). These mRNA-specific complementary oligonucleotides bear both a fluorophore and a quencher; the binding of a beacon to its targeted mRNA separates the fluorophore and quencher, resulting in a fluorescent signal that can be tracked in live cells. Similar probes have recently been used to track endogenous *β-actin* in *Xenopus* axons (32), yielding dynamic properties similar to those observed in vivo using the MS2–*β-actin* mouse (25).

In living primary rat hippocampal neuronal cultures (day in vitro [DIV] 21+) molecular beacons targeting endogenous *Camk2α*, *β-actin*, or *Psd95* mRNA (*Materials and Methods*) were imaged to report on endogenous mRNA dynamics for up to 20 min (Movies S1–S3). A nontargeting GFP probe showed no specific signal in the soma or dendrites (Movie S4). Interestingly, we observed a heterogenous size distribution for the mRNA puncta, with larger pronounced particles seen near the soma (Movies S1–S3), similar to what has been reported previously for *β-actin* (25). In addition, we detected a number of apparent dendritic mRNA–mRNA fusion events (*SI Appendix*, Fig. S1*A* and Movie S5), suggesting that these mRNAs can exist in a heterogenous copy number state, in addition to proposed modes of single mRNA transport in axons and dendrites (29, 32, 33). To assess this quantitatively, we analyzed the intensity of the individual beacon puncta (i.e., mRNA granules) and compared it with a commercially synthesized standard containing a single ATTO647N fluorophore anchored on a glass coverslip (*Materials and Methods*). Using this standard, we determined that a sizeable fraction of each mRNA exhibited an intensity consistent with a single mRNA molecule (Fig. 1 *B* and *C* and *SI Appendix*, Fig. S1 *A*–*C*). *β-Actin* mRNAs, in particular, were often detected in a range consistent with a single copy number state (Fig. 1*C* and *SI Appendix*, Fig. S1 *A*–*C*), in line with previous reports (29, 32). Interestingly, while both *Camk2a* and *Psd95* mRNAs also were detected as single copy granules, a noticeable fraction of each population existed in a multimeric state (Fig. 1*C* and *SI Appendix*, Fig. S1 *A*–*C*), indicating that higher-order (containing more than a single mRNA) mRNA granules exist within the dendrite. To validate this, we performed single molecule FISH (smFISH) (*Materials and Methods*). Assessing the intensity of RNA puncta in the dendrite, we found a wider range of intensities for *Camk2a* and *Psd95* compared with *β-actin*, consistent with a multimeric copy number state (Fig. 1 *D* and *E* and *SI Appendix*, Fig. S1*D*). Taken together, these data suggest that while the majority of these dendritically localized mRNAs exists in a single copy number state, higher-order multimeric states exist and may be a transcript-specific feature, likely determined through specific sets of RBPs bound to particular mRNAs.

**Fig. 2.**
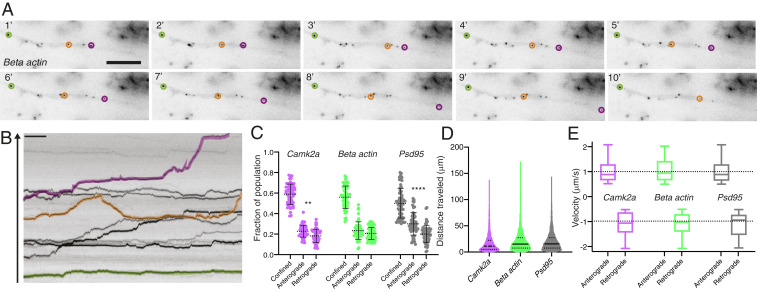
Tracking and classifying mRNA dynamics along dendrites in live neurons. (*A*) Still images from a *β-actin* Atto647N-labeled dendrite (Movie S6), shown are single frames every minute for 10 min (of 20 total). Individual mRNA puncta are highlighted to illustrate distinct dynamic profiles, mainly stationary (green) or confined with periods of motility (orange and magenta). (Scale bar, 5 μm.) (*B*) Kymograph from the first 10 min of (Movie S6). Arrow denotes the anterograde direction along the dendrite. (Scale bar, 1 min.) (*C*) Quantification of mRNA dynamic state: confined, anterograde vs. retrograde for Atto565 *Camk2a*, Atto565 *β-actin*, and Atto565 *Psd95*. *n* = 59 cells. **P < 0.01, ****P < 0.0001 Holm–Sidak’s multiple comparison test. (*D*) Quantification of cumulative distance traveled for Atto565 *Camk2a*, Atto565 *β-actin*, and Atto565 *Psd95. n* = 59 cells. (*E*) Quantification of mRNA velocity for anterograde and retrograde for Atto565 *Camk2a* (1.013 ± 0.450; −1.083 ± 0.480), Atto565 *β-actin* (1.054 ± 0.493; −1.110 ± 0.452), and Atto565 *Psd95* (0.997 ± 0.426; −1.111 ± 0.479). *n* = 59 cells.

To capture the dynamic behavior of each mRNA species within dendrites, we employed a semiautomated tracking approach. Using a custom-written analysis pipeline (*Materials and Methods*) we quantified the beacon mRNA dynamics (Fig. 2 *A*–*C*), cumulative distance traveled (Fig. 2*D*), and transport velocity (Fig. 2*E*) for all three mRNA targets. To assess mRNA dynamics, we measured the percentage of time (during the entire imaging epoch) a detected granule spent actively moving within the dendrite (either anterogradely: away from the cell body; or retrogradely: toward the cell body) or exhibited a confined behavior. For all experiments we acquired images at one frame per second for up to 20 min (*Materials and Methods*). “Confined” behavior was assigned to periods of time when an mRNA granule exhibited restricted (<0.5 μm) movement within the dendrite (*Materials and Methods*).

Similar to previous reports (22, 32), we found that all three mRNAs spent most of their time in a confined state (fraction population; *Camk2a*: 0.59 ± 0.10; *β-actin*: 0.56 ± 0.11; *Psd95*: 0.50 ± 0.14; mean fraction of time ± SEM). For active movement all three mRNAs displayed a slight bias for anterograde transport (fraction population, anterograde vs. retrograde: *Camk2a*: 0.23 ± 0.06 vs. 0.18 ± 0.07; *β-actin*: 0.23 ± 0.09 vs. 0.22 ± 0.06; *Psd95*: 0.31 ± 0.11 vs. 0.20 ± 0.09) (Fig. 2*A*), explaining how mRNAs can eventually populate more distal regions of the dendrite. Interestingly, *Psd95* granules exhibited enhanced motility (less in the confined state) (Fig. 2*C*) compared with *β-actin* and *Camk2α*. All three mRNAs traveled similar distances (∼20 μm on average) over the imaging epoch and exhibited similar velocities (∼1 μm/s) for both anterograde and retrograde transport, consistent with the mixed polarity of microtubules within the dendrite (34). We noted that while the majority of mRNA molecules we measured (orange and magenta highlighted puncta in Fig. 2 *A* and *B*) alternated between periods of confined vs. active transport, a small fraction of particles (green in Fig. 2 *A* and *B*) showed little to no active transport during the entire imaging session. We therefore further distinguished “confined” vs. truly “stationary” events (*Materials and Methods* and *SI Appendix*, Fig. S2*A*) and found ∼6% of the confined population were better characterized as stationary events. In all subsequent analyses, we removed these stationary events from the analysis.

### Translational Inhibition Alters mRNA Dynamics within the Dendrite.

**Fig. 3.**
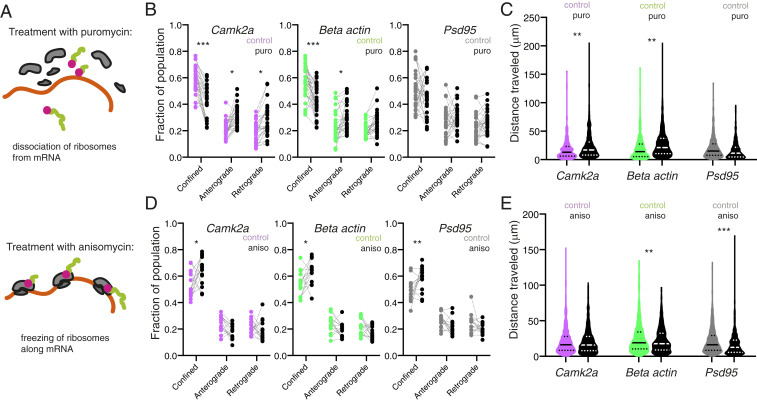
Manipulating ribosome association of mRNAs results in transcript-specific alterations in mRNA dynamics. (*A*) Schematic representation of the effects of puromycin (*Upper*) or anisomycin (*Lower*) on ribosomal association with mRNA. Puromycin results in ribosomal subunit disassembly, whereas anisomycin results in stalling of elongating ribosomes. (*B*) Quantification of mRNA dynamic state: confined, anterograde vs. retrograde for Atto647N *Camk2a*, Atto647N *β-actin*, and Atto647N *Psd95* for control vs. puro treated samples. *n* = 29 cells per condition. *P < 0.05; ***P < 0.001. Paired *t* test. (*C*) Quantification of cumulative distance traveled for Atto647N *Camk2a*, Atto647N *β-actin*, and Atto647N *Psd95* for control vs. puro-treated samples. *n* = 29 cells per condition. **P < 0.01 Sidak’s multiple comparisons test. (*D*) Quantification of mRNA dynamic state: confined, anterograde vs. retrograde for Atto647N *Camk2a*, Atto647N *β-actin*, and Atto647N *Psd95* for control vs. anisomycin-treated samples. *n* = 15 cells per condition. *P < 0.05; **P < 0.01 paired *t* test. (*E*) Quantification of cumulative distance traveled for Atto647N *Camk2a*, Atto647N *β-actin*, and Atto647N *Psd95* for control vs. anisomycin-treated samples. *n* = 15 cells per condition. **P < 0.01; ***P < 0.001 Sidak’s multiple comparisons test.

With the above measurements of basal mRNAs dynamics, we next assessed if we could alter their dynamic properties. We first assessed if perturbing the translational status of an mRNA could affect its motility. To alter the translation status of an mRNA, we used two mechanistically distinct translational elongation inhibitors (Fig. 3*A*): puromycin, which causes release of the nascent peptide chain and ribosomal dissociation from the mRNA (35), and anisomycin, which freezes elongating ribosomes on mRNAs (36). As such, puromycin promotes the transition to a ribosome-free mRNA state, whereas anisomycin causes ribosome accumulation on mRNAs. Using our analysis pipeline, we quantified the effects of these treatments on mRNA dynamics (Fig. 3 *B*–*E*). For *Camk2α* and *β-actin*, puromycin displacement of ribosomes led to enhanced mRNA motility (reduced confinement) (fraction population confined; *Camk2a*: 0.59 ± 0.10 vs. 0.43 ± 0.13; *β-actin*: 0.56 ± 0.13 vs. 0.44 ± 0.12; mean ± SEM) (Fig. 3*B*) and cumulative distance traveled (distance in micrometers; *Camk2a*: 18.14 ± 20.26 vs. 24.48 ± 25.70; *β-actin*: 19.64 ± 20.73 vs. 27.59 ± 24.78) (Fig. 3*C*). In contrast, *Psd95* motility (fraction population confined; *Psd95*: 0.51 ± 0.14 vs. 0.45 ± 0.14) (Fig. 3*B*) and cumulative distance traveled (Fig. 3*C*) (distance in micrometers; *Psd95*: 20.71 ± 19.21 vs. 17.44 ± 20.41) was not significantly changed by puromycin treatment. This difference may reflect a higher basal translational state for *Camk2α* and *β-actin* mRNAs, consistent with *Psd95* mRNA being slightly more dynamic relative to the other two mRNAs (Fig. 2).

Since displacing ribosomes for *Camk2α* and *β-actin* enhanced mRNA motility, we predicted that freezing the ribosomes on the mRNA should lead to the opposite effect. Indeed, anisomycin treatment led to decreased mRNA motility (Fig. 3*D*) for all three mRNAs (fraction population confined; *Camk2a*: 0.54 ± 0.10 vs. 0.64 ± 0.11; *β-actin*: 0.55 ± 0.09 vs. 0.63 ± 0.09; *Psd95*: 0.49 ± 0.09 vs. 0.58 ± 0.09; mean ± SEM) and cumulative distance traveled (Fig. 3*E*) for *β-actin* and *Psd95* (distance in micrometers; *Camk2a*: 21.39 ± 19.24 vs. 20.06 ± 17.85; *β-actin*: 25.65 ± 22.24 vs. 22.32 ± 17.66; *Psd95*: 21.47 ± 17.70 vs. 16.96 ± 16.75). Interestingly, neither displacing nor freezing ribosomes had an effect on the active transport velocity of any mRNA (*SI Appendix*, Fig. S2 *B* and *C*). Furthermore, neither puromycin nor anisomycin affected the stationary population (*SI Appendix*, Fig. S2 *D* and *E*). Given that transport velocity was unaffected by either perturbation of translation (*SI Appendix*, Fig. S2 *B* and *C*), our data are consistent with the idea that mRNAs are transported in a quiescent nontranslating state (23, 37). Taken together, these data illustrate that the translational status of a given mRNA will affect its dynamics within the dendrite.

### Plasticity Stalls mRNA Transport and Accumulates mRNAs near Dendritic Spines.

**Fig. 4.**
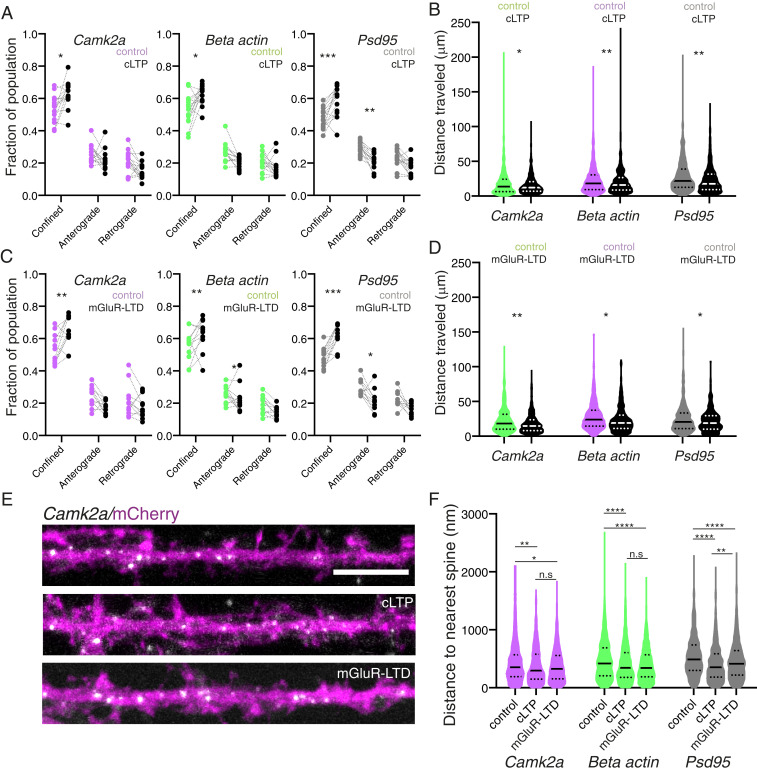
Two distinct forms of synaptic plasticity attenuate mRNA dynamics near dendritic spines. (*A*) Quantification of mRNA dynamic state: confined, anterograde vs. retrograde for Atto647N *Camk2a*, Atto647N *β-actin*, and Atto647N *Psd95* for control vs. cLTP induced samples. *n* = 14 cells per condition. *P < 0.05; **P < 0.01; ***P < 0.001. Paired *t* test. (*B*) Quantification of cumulative distance traveled for Atto647N *Camk2a*, Atto647N *β-actin*, and Atto647N *Psd95* for control vs. cLTP induced samples. *n* = 14 cells per condition. *P < 0.05; **P < 0.01. Sidak’s multiple comparisons test. (*C*) Quantification of mRNA dynamic state: confined, anterograde vs. retrograde for Atto647N *Camk2a*, Atto647N *β-actin*, and Atto647N *Psd95* for control vs. mGluR-LTD induced samples. *n* = 14 cells per condition. *P < 0.05; **P < 0.01; ***P < 0.001. Paired *t* test. (*D*) Quantification of cumulative distance traveled for Atto647N *Camk2a*, Atto647N *β-actin*, and Atto647N *Psd95* for control vs. mGluR-LTD–induced samples. *n* = 14 cells per condition. ; *P < 0.05; **P < 0.01. Sidak’s multiple comparisons test. (*E*) Example images of Camk2a RNA FISH signal in neurons volume filled with mCherry under control, +cLTP and +mGluR-LTD conditions. (Scale bar, 2.5 μm.) (*F*) Quantification of mRNA distance to the nearest spine reveals a slight decrease in the distance for all three mRNAs during plasticity. Control, cLTP, mGluR-LTD ± SD: *Camk2a* (411.6 ± 300.7; 376.8 ± 292.5; 375.0 ± 275.4), *β-actin* (474.3 ± 327.1; 413.8 ± 312.3; 391.0 ± 275.8), and *Psd95* (537.2 ± 334.3; 403.3 ± 283.7; 454.6 ± 304.7) *P < 0.05; **P < 0.01; ****P < 0.0001; n.s., not significant. Dunn’s multiple comparisons test. *n* = 15 cells per condition.

We next assessed if we could modulate mRNA dynamics with physiologically relevant manipulations, specifically synaptic plasticity. We examined how mRNA dynamics were altered during two forms of protein synthesis-dependent plasticity, cLTP (38) and mGluR-LTD (39) (*SI Appendix*, Fig. S3 *A* and *B*). cLTP induction (*Materials and Methods*) was performed by a 5-min incubation in magnesium-free buffer supplemented with glycine and picrotoxin, whereas as mGluR-LTD was induced with 100 μM (S)-3,5-Dihydroxyphenylglycine hydrate (S-DHPG) for 5 min. Following cLTP (Fig. 4 *A* and *B* and *SI Appendix*, Fig. S3*C*) or mGluR-LTD (Fig. 4 *C* and *D* and *SI Appendix*, Fig. S3*D*) mRNA dynamics were monitored immediately after agonist washout. Induction of cLTP led to decreased mRNA motility (Fig. 3*D*) for all three mRNAs (fraction population confined; *Camk2a*: 0.54 ± 0.09 vs. 0.63 ± 0.08; *β-actin*: 0.53 ± 0.10 vs. 0.62. ± 0.06; *Psd95*: 0.47 ± 0.06 vs. 0.58 ± 0.10; mean ± SEM) and reduced cumulative distance traveled (Fig. 4*B*) (total cumulative distance traveled in microns: *Camk2a*: 23.36 ± 19.86 vs. 20.54 ± 18.97; *β-actin*: 18.95 ± 19.75 vs. 16.25 ± 14.52; *Psd95*: 28.95 ± 23.29 vs. 22.87 ± 18.91). Induction of mGluR-LTD led to decreased mRNA motility (Fig. 4*C*) for all three mRNAs (fraction population confined; *Camk2a*: 0.54 ± 0.09 vs. 0.64 ± 0.09; *β-actin*: 0.56 ± 0.10 vs. 0.63 ± 0.10; *Psd95*: 0.48 ± 0.06 vs. 0.62 ± 0.07; mean ± SEM) and reduced cumulative distance traveled (Fig. 4*D*) (total cumulative distance traveled in microns: *Camk2a*: 28.81 ± 20.55 vs. 23.66 ± 18.48; *β-actin*: 23.37 ± 19.34 vs. 17.97 ± 14.00; *Psd95*: 25.54 ± 20.68 vs. 21.84 ± 16.05). To summarize, we observed a significant decrease in the time all three mRNAs spent moving and a reduced cumulative distance traveled within the dendrite following induction of both forms of plasticity.

To assess more precisely the location of mRNA deposition during these enhanced periods of mRNA confinement, we performed high-resolution smFISH in dendrites immediately after induction of cLTP or mGluR-LTD (*Materials and Methods* and Fig. 4 *E* and *F*). We measured the mean distance of an mRNA granule to its nearest dendritic spine and found that this distance decreased significantly with both cLTP and mGluR-LTD induction for all three mRNAs (Fig. 4*F*). Taken together with the altered dynamics observed with the molecular beacons (Fig. 4 *A* and *C*), our data suggest increased spine association of these mRNAs during plasticity. This enhanced association may fuel local translation of these mRNAs to induce and maintain both forms of structural plasticity.

### Exploring the Dynamics of Protein Synthesis in Real Time.

**Fig. 5.**
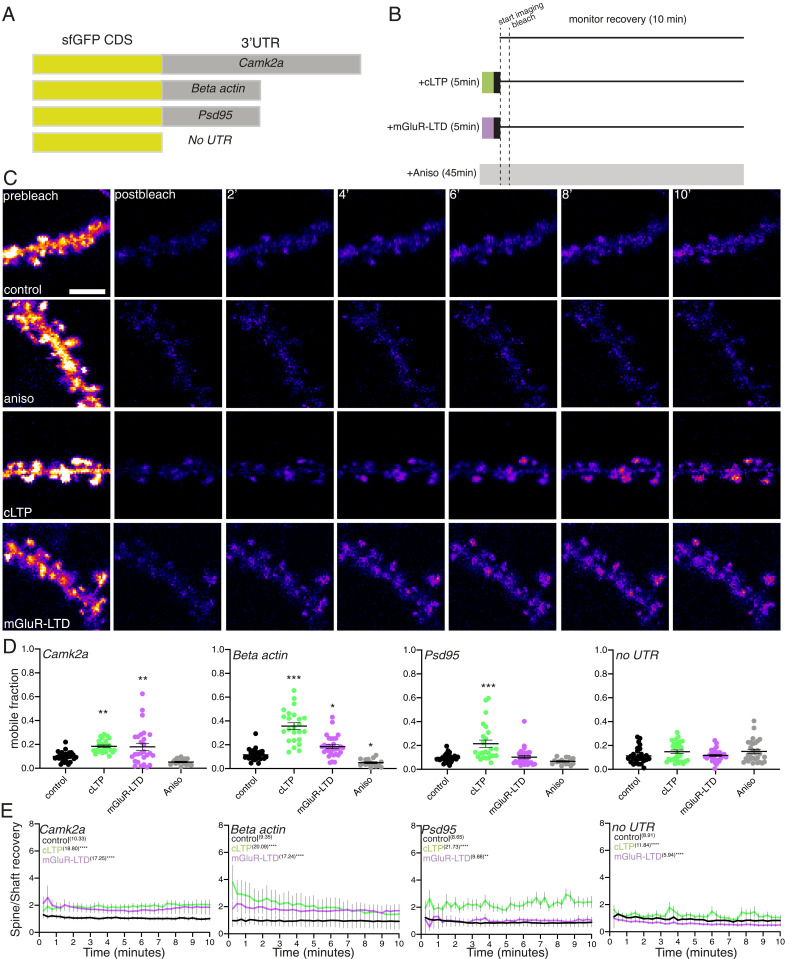
Real-time visualization of 3′UTR regulated translation during synaptic potentiation and depression. (*A*) Scheme for the reporters used to assess real-time 3′UTR regulated translational dynamics in live neurons. (*B*) Scheme of workflow for the treatment and visualization of plasticity regulated protein synthesis. Following induction of cLTP or mGluR-LTD, the indicated pharmacological treatment was washed out (black box) and the samples were then imaged for 2 min every 15 s to acquire a baseline measurement. The cells were then bleached and the fluorescence recovery was monitored every 15 s for 10 min. For controls, either no treatment or treatment with the translational inhibitor anisomycin was used for comparison. (*C*) Example image of a Camk2a sfGFP reporter under control (top row) +anisomycin (second row), +cLTP (third row), or +mGluR-LTD conditions before bleaching and during the phase of fluorescence recovery. (Scale bar, 5 μm.) (*D*) Mobile fractions calculated for the translational reporters during control, plasticity induction and anisomycin treatment. *P < 0.05; **P < 0.01; ***P < 0.001. Dunnett’s multiple comparisons test for treated vs. control condition for each construct; *n* = >14 cells per condition. (*E*) Recovery rate of fluorescence of dendritic spines to shafts demonstrates a bias for spine fluorescence recovery during plasticity. Kruskal-Wallis test. **P < 0.01; ****P < 0.0001. *n* = >14 cells per condition.

To assess directly whether translation of these three mRNAs was altered during cLTP or mGluR-LTD, we used translational reporters (18) (Fig. 5) comprising a codon optimized superfolder GFP (40) (sfGFP) (*Materials and Methods*) flanked by the corresponding dendritically-enriched 3′UTR (41) of *Cam2a*, *β-actin*, or *Psd-95*. Each 3′UTR was included to confer both transcript-specific localization and translational regulation to the translational reporter (41). We used cell-wide fluorescence recovery after photobleaching (FRAP) to visualize newly synthesized proteins. Following whole-cell photobleaching, we measured the emergence and time course of the protein synthesis-dependent fluorescence signal to assess the kinetics and extent of the translational responses for each mRNA. We found that all three transcript-specific reporters showed protein synthesis-dependence in their recovery compared with the no UTR control (Fig. 5*D* and *SI Appendix*, Fig. S4, black vs. gray curves), indicating that these reporters are effective readouts for active translation. The induction of either cLTP or mGluR-LTD resulted in an enhancement of the mobile fraction (Fig. 5*D*) and total fluorescence recovery (*SI Appendix*, Fig. S4) for both the CAMK2α and β-ACTIN reporter, indicating enhanced protein synthesis. Conversely, PSD-95 showed enhanced translation following cLTP induction (Fig. 5*D* and *SI Appendix*, Fig. S4), but no change following mGluR-LTD. Interestingly, we noticed a strong bias for the emergence of fluorescence in spines during cLTP and mGluR-LTD (Fig. 5*C*). To assess if this represented a bias for spine accumulation of newly synthesized protein, we assessed the ratio of fluorescence recovery for the spine over the shaft (Fig. 5*E*). Plasticity induction indeed resulted in a higher rate of spine recovery, except for PSD-95 during mGluR-LTD, suggesting that protein production near spines could drive the emergence of new fluorescence in the spines. Taken together, these data indicate that there can be a functional disconnect between transcript-specific changes in mRNA dynamics seen during plasticity (Fig. 3) and the downstream translational state of that mRNA species.

**Fig. 6.**
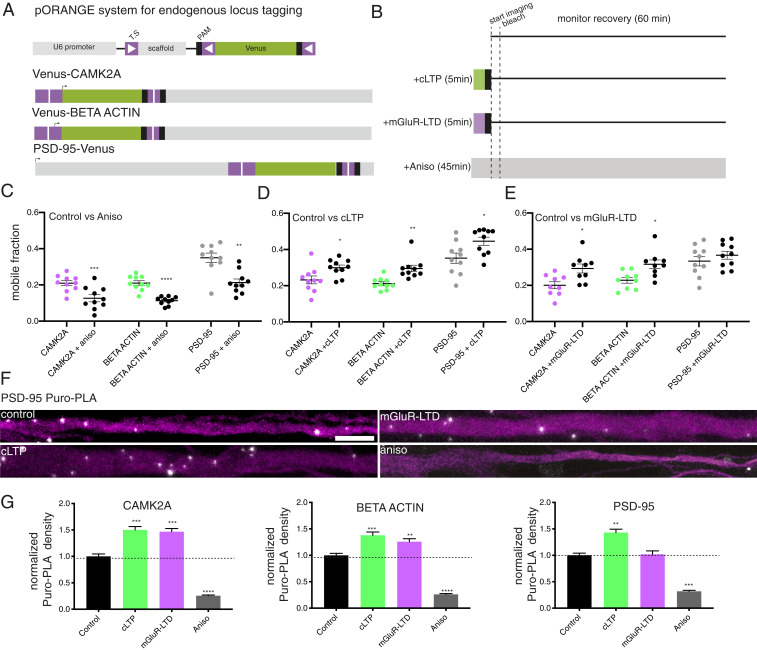
Visualizing endogenous protein translation in real-time during synaptic potentiation and depression. (*A*) Scheme for the modification of endogenous gene tagging of *Camk2a/β-actin/Psd95* with Venus fluorescent protein. T.S.; targeting sequence; arrow indicates start codon. (*B*) Scheme of workflow for the treatment and visualization of plasticity regulated protein synthesis. Following induction of cLTP or mGluR-LTD, the pharmacological treatment was washed out (black box) and the samples were then imaged for 2 min every 15 s to acquire a baseline measurement. The cells were then bleached and the fluorescence recovery was then monitored every 15 s for 60 min. For controls, either no treatment or treatment with the protein synthesis inhibitor anisomycin was used for comparison. (*C*) Mobile population during the FRAP recovery for Venus-CAMK2a, Venus–β-ACTIN, and PSD-95–Venus during control and anisomycin treatment. *n* = 10 cells per condition. Two-tailed paired *t* test. **P < 0.01; ***P < 0.001; ****P < 0.0001. (*D*) Mobile population during the FRAP recovery for Venus-CAMK2a, Venus–β-ACTIN, and PSD-95–Venus during control and cLTP. *n* = 10 cells per condition. Two-tailed paired *t* test. *P < 0.05; **P < 0.01. (*E*) Mobile population during the FRAP recovery for Venus-CAMK2a, Venus–β-ACTIN, and PSD–95-Venus during control and mGluR-LTD. *n* = 9 to 10 cells per condition. Two-tailed paired *t* test. *P < 0.05. (*F*) Example images dendritically localized (MAP2, magenta) Puro-PLA signal for PSD-95 (white) under control and stimulated conditions. (Scale bar, 10 μm.) (*G*) Puro-PLA quantification reveals local protein synthesis underlies the translational responses of CAMK2a, β-ACTIN, and PSD-95 during cLTP and mGluR-LTD. *n* = 85 cells per condition. Dunnett’s multiple comparisons test. **P < 0.01; ***P < 0.001; ****P < 0.0001.

To assess if the same pattern of translation following plasticity was also observed with the endogenous transcripts, we used CRISPR/Cas9 gene editing in neurons (42) to tag endogenous CAMK2α or β-ACTIN (N terminal) or PSD-95 (C terminal) protein with the fast-folding Venus fluorescent protein (43) (Fig. 6*A* and *SI Appendix*, Fig. S4). The fast-folding nature of Venus (*t*_1/2__maturation_ = 2 to 5 min) allowed us to rapidly assess the translational regulation of these three proteins. All three proteins were successfully tagged and exhibited their characteristic localization patterns (*SI Appendix*, Fig. S5). Venus-tagged CAMK2α and β-ACTIN were enriched in axons and dendrites, and most strongly enriched in dendritic spines and axonal boutons, while PSD-95–tagged Venus was exclusively enriched within postsynaptic compartments. As before, we performed cell-wide FRAP (*Materials and Methods*, Fig. 6 *B*–*E*, and *SI Appendix*, Fig. S6 and Table S2). We tracked the emergence and time course of the protein synthesis-dependent fluorescence signal to assess the kinetics and the extent of the translational responses for each mRNA (*SI Appendix*, Fig. S6 *B*–*D*). For all proteins, treatment with the protein synthesis inhibitor anisomycin significantly reduced the emergence of new fluorescent signal (*SI Appendix*, Fig. S6*B*) and attenuated the mobile population during recovery (Fig. 6*C*) (fraction mobile population; CAMK2α: 0.21 ± 0.01 vs. 0.13 ± 0.06; β-ACTIN: 0.21 ± 0.01 vs. 0.11 ± 0.007; PSD-95: 0.35 ± 0.03 vs. 0.21 ± 0.02; mean ± SEM), confirming our ability to visualize new protein synthesis in real-time for endogenous proteins.

In line with our reporter observations, we found that cLTP induction significantly enhanced the emergence of fluorescence for all three proteins (*SI Appendix*, Fig. S6*C*) and enhanced the mobile population (Fig. 6*D*) (fraction mobile population; CAMK2α: 0.23 ± 0.02 vs. 0.3 ± 0.01; β-ACTIN: 0.21 ± 0.01 vs. 0.30 ± 0.02; PSD-95: 0.35 ± 0.03 vs. 0.45 ± 0.02; mean ± SEM), indicating enhanced protein synthesis. Induction of mGluR-LTD, however, elicited a transcript-specific enhancement of protein synthesis for CAMK2α and β-ACTIN but not PSD-95 (fraction mobile population; CAMK2α: 0.20 ± 0.02 vs. 0.29 ± 0.02; β-ACTIN: 0.23 ± 0.02 vs. 0.32 ± 0.02; PSD-95: 0.33 ± 0.03 vs. 0.37 ± 0.02; mean ± SEM), (Fig. 6*E* and *SI Appendix*, Fig. S6*D*). In the above experiments, a brief (5 min) application of DHPG did not enhance the synthesis of PSD-95. Prior studies (16, 17, 44) have shown that a sustained application of the DHPG can stimulate PSD-95 translation. To assess the effect of a longer period of mGluR activation, we treated neurons for with DHPG throughout the FRAP experiment (∼60 min) (*SI Appendix*, Fig. S7 *A* and *B* and Table S3). We found that the mobile fraction of PSD-95 Venus was enhanced by the long duration DHPG application (*SI Appendix*, Fig. S7*B*), and this effect was blocked by pretreatment with anisomycin. We found no change in the CAMK2a or β-ACTIN (*SI Appendix*, Fig. S7*C*) mobile fraction. These data are consistent with PSD-95 translation regulation by long-term but not brief (mGluR-LTD) mGluR activation.

To obtain better temporal and spatial resolution on the translational responses to plasticity induction, we validated the above findings with a method that couples general metabolic labeling of nascent proteins (puromycin) with a specific label for a protein-of-interest using the proximity ligation assay (Puro-PLA) (45) (*Materials and Methods* and *SI Appendix*, Fig. S8*A*). We examined changes in dendritic protein synthesis during plasticity (Fig. 6 *F* and *G* and *SI Appendix*, Fig. S8 *B* and *C*). Consistent with previous observations (45), we observed that there was a protein synthesis dependence to the Puro-PLA signal for all three proteins (black vs. gray bars in Fig. 6*G*) (normalized PLA density; CAMK2α: 0.26 ± 0.13; β-ACTIN: 0.26 ± 0.14; PSD-95: 0.32 ± 0.17; mean ± SEM). Furthermore, cLTP induction enhanced the dendritic synthesis of all three mRNAs (normalized PLA density; CAMK2α: 1.50 ± 0.58; β-ACTIN: 1.38 ± 0.57; PSD-95: 1.43 ± 0.58; mean ± SEM), while induction of mGluR-LTD only enhanced the synthesis of CAMK2α and β-ACTIN (normalized PLA density; CAMK2α: 1.47 ± 0.55; β-ACTIN: 1.26 ± 0.52; PSD-95: 1.02 ± 0.61; mean ± SEM). These data not only validate and support our real-time translational response observations, but also support that this translation can occur locally within the dendrite where we detect changes in mRNA dynamics. Taken together, these data indicate that alterations in mRNA dynamics and protein synthesis underlie the manifestation of specific forms of synaptic plasticity.

## Discussion

Here we investigated the interplay between mRNA dynamics and translation within neuronal dendrites during two different forms of synaptic plasticity. We characterized the dynamics and translation of three individual endogenous mRNAs: *Camk2a*, *β-actin*, and *Psd95*, during basal neuronal activity and plasticity. In live hippocampal neurons we provide evidence that mRNAs exist in heterogenous copy number organizational states (Fig. 1). The preference for single mRNA copy vs. higher-order state may be a transcript-specific feature, as *Camk2a* and *Psd95* exhibit an enhanced preference for higher-order multimeric states compared with the previously described single mRNA state of *β-actin* (29). We found that, on average, different mRNA transcripts displayed remarkably similar dynamic properties (velocity, time spent motile, distance traveled) within the dendrite. Only subtle differences were seen, with *Psd95* showing a higher fraction of time spent motile than *β-actin* or *Camk2a*. The factors influencing *Psd95*’s higher motility remain to be elucidated, but may reflect a difference in its translational efficiency (46) as an FMRP-regulated transcript (17, 30). We found that ribosome association directly influences mRNA dynamics, suggesting that mRNA translation is likely restricted to nontransporting mRNAs exclusively within the dendrite. Consistent with our data, previous work in fibroblasts (47) and axons (32) has shown that diffusion of *β-actin* increases when ribosomes are displaced. These data fit with recent proteomic analysis on isolated mRNA transport granules (48), which detected only a subset of ribosomal proteins associated with granules.

In our experiments, mRNAs were sequestered, exhibiting reduced mobility following plasticity. The mechanisms underlying the initial capture and enduring sequestration of mRNAs are not well understood. Recent work with *Rgs4* indicated that neuronal activity is essential for the capture of mRNAs at dendritic spines; inhibition of neuronal activity with TTX suppressed mRNA-spine association (24). An activity dependence for spine capture has also been reported with *β-actin* during glutamate uncaging (22). This was proposed to be dependent on underlying actin remodeling from the structural plasticity induced by the glutamate uncaging. Indeed, the structural spine plasticity characteristic of both cLTP and mGluR-LTD involves modulation of the actin cytoskeleton (49). Whether actin remodeling broadly promotes mRNA sequestration at or near dendritic spines remains to be assessed. The translation of all three mRNAs was enhanced by cLTP, but mGluR-LTD only enhanced the translation of *Camk2a* and *β-actin *indicating mRNA sequesteration alone does not lead to translation. Taken together, our data dissociate the accumulation and association of mRNAs near synapses from their translational status.

*Psd95* mRNA has previously been characterized as an mGluR-regulated transcript (16, 30, 44); however, following washout of the mGluR agonist (similar to the conditions used here), PSD-95 protein is rapidly degraded (50). This functional switch from promoting PSD-95 mRNA translation to degrading PSD-95 protein might be an essential step for the manifestation of spine shrinkage in mGluR-LTD. Activation of mGluR signaling has previously been linked to phosphorylation of eIF2a, promoting the translation of transcripts with 5′UTR containing upstream open reading frames (uORFs) (51). During the long-term DHPG treatment, we observed an enhancement of PSD-95 protein synthesis (*SI Appendix*, Fig. S7*B*); this may be similarly dependent on eIF2a phosphorylation. After the DHPG is washed out, and presumably eIF2a returns to a nonphosphorylated state, then PSD-95 translation may return to baseline levels. A similar functional switch in translational output of *Psd95* mRNA has been previously described for FMRP-regulated translation in mGluR signaling (17, 30). We note that currently available methods for the induction of mGluR-dependent mGluR-LTD are limited to bath application of mGluR-agonists; the development of a caged mGluR agonists would enable the analysis of single spines.

How mRNAs associated with activated synapses become translational competent likely depends on signaling cascades underlying various forms of synaptic plasticity. Given that specific signaling cascades are turned on by distinct forms of plasticity (2), these cascades likely influence changes in posttranslational modifications of RBPs on particular transcripts, regulating their “translatability.” Consistent with this, activation of PKA signaling alone is sufficient to enhance dendritic protein synthesis (11). cLTP, known classically for its dependence on CamkII signaling (52), also triggers a number of other classic signaling cascades, including PKA (53), PKC (54, 55), MAPK/ERK (56), PI3K (57), mTOR (58), and Src (59). mGluR-LTD on the other hand, is less clear in its signaling requirements; it involves activation of PKC (60) and PI3K/AKT/mTOR (61). However, the role of CamkII (62, 63) is debated. Once sequestered by an active synapse, mRNAs could be “synaptically decoded” by the activation of these signaling pathways to determine whether and when a given mRNA species will be translated or not.

## Materials and Methods

### Molecular Beacon Structure and Design.

#### Solid-phase synthesis.

Milli-Q water was treated with DEPC (0.1%) overnight and autoclaved.

The following oligonucleotides were synthesized on an ABI392 instrument:

PSD95_1: 5′- M CAC​GAC​CAU​CCC​UCC​CCU​UUU​CCC​AAA​AAA​AUA​UCG​UG Q_1_ -3′
PSD95_2: 5′- M CAC​GAA​UAA​AAU​CCC​AGA​AAA​AAA​AAA​AGC​CUC​GUG Q_2_ S -3′;
CAMK2_1: 5′- M CAC​GAG​GUA​AAA​ACU​UCC​CCU​CAC​UCC​UCU​UCC​UCG​UG Q_1_ -3′;
CAMK2_2: 5′- M CAC​GAU​UUU​UCU​UCU​UUU​UUG​UUU​UGC​UCU​UCG​UG Q_2_ S -3′;
β-Actin_1: 5′- M CAC​GAC​AAA​ACA​AAA​CAA​AAA​AAC​UUA​AAA​AAA​UCG​UG Q_1_ -3′;
β-Actin_2: 5′- M CAC​GAU​UCA​CCG​UUC​CAG​UU UUUAAAUCCUGUCGUG Q_2_ S -3′;
GFP_1: 5′- M CAC​GAA​CUU​CUU​CAA​GUG​UGC​GAU​GCC​AGA​AGG​GUC​GUG Q_1_ -3′;
M = Fmoc-Amino-DMT C-3 CED phosphoramidite (ChemGenes);
Q_1_ = 3′-BHQ-2 CPG 1000 (LinkTech);
Q_2_ = BBQ-650-(DMT)-CE-Phosphoramidite (LinkTech);
S = 3′-Spacer C3 SynBase CPG 1000/110 (LinkTech);
A = 2′-OMe-Pac-A-CE Phosphoramidite (LinkTech);
C = 2′-OMe-Ac-C-CE Phosphoramidite (Linktech);
G = 2′-OMe-*i*Pr-Pac-G-CE Phosphoramidite (LinkTech);
U = 2′-OMe-U-CE Phosphoramidite (LinkTech).

For all synthesized oligonucleotides, Pac_2_O was used as capping reagent. 0.3 M BTT (emp Biotech) was used as activator. Coupling time for A, C, G, U, and M was 6 min, Q_2_ for 15 min. Synthesis was performed in DMTr-On mode. The cyanoethyl groups were removed by flushing the columns with 20% diethylamine (emp Biotech) for 10 min, followed by washing with MeCN, Argon, and drying in vacuum. Cleavage from the solid-phase was performed with aqueous ammonia (32%) (Merck) for 4 h at room temperature. After spin filtration, the solvent was removed at 4 °C using a vacuum concentrator (SpeedVac, Thermo Fischer).

#### Purification.

The DMTr-On oligonucleotides were purified by RP-HPLC on an Agilent 1200 equipped with a Waters XBridge BEH C18 OBD column (300 Å, 5 µm, 19 × 250 mm, 4 mL/min, 60 °C). As solvents 400 mM hexafluoroisopropanol (Fluorochem), 16.3 mM Et_3_N (Merck), pH 8.3, and MeOH (Fluka) were used with a gradient from 5 to 100% MeOH in 30 min. After separation, the solvent was evaporated in a vacuum concentrator at 4 °C. The DMTr group was removed by incubation of the oligonucleotides in 400 µL 80% aqueous AcOH (Merck) at room temperature for 20 min, followed by evaporating the solvent in a vacuum concentrator at 4 °C. The RNAs were again purified by RP-HPLC under the same conditions as above.

#### Fluorophore labeling.

For fluorophone labeling, 10 nmol of each RNA were dissolved in 150 µL borate-buffer (0.1 M sodium tetraborate (Merck), pH 8.4). GFP_1, PSD95_1, CAMK2_1 and β-actin_1 were incubated with 200 nmol ATTO565 NHS (ATTO-TEC), dissolved in 50 µL DMF (Lumiprobe, labeling grade), for 4 h at 37 °C. PSD95_2, CAMK2_2 and Beta Actin_2 were incubated with 200 nmol ATTO647N NHS (ATTO-TEC), dissolved in 50 µL DMF, for 4 h at 37 °C. Buffer and the excess of fluorophore were removed by size-exclusion chromatography (NAP 25, GE Healthcare). The solvent was evaporated at 4 °C using a vacuum concentrator. The residue was purified by RP-HPLC on an Agilent 1200 equipped with an Xbridge BEH C18 OBD (300 Å, 3.5 µm, 4.6 × 250 mm, 1 mL/min, 60 °C). As solvents 400 mM hexafluoroisopropanol, 16.3 mM Et_3_N, pH 8.3 and MeOH were used with a gradient from 5% MeOH to 100% MeOH in 50 min.

#### Sample preparation for in vivo use.

For use in living cells, the remaining HPLC buffer ions had to be removed. Therefore, the oligonucleotides were dissolved in 0.3 M NaOAc (Merck) (10 µL per 1 nmol RNA). EtOH (Fluka, prechilled to −20 °C, 40 µL per 1 nmol RNA) was added. The mixture was cooled to −20 °C for at least 6 h. The precipitant was pelletized by centrifugation at 4 °C, 20,000 × *g* for 20 min. The residue was redissolved in 0.3 M NaOAc and the precipitation steps were repeated three times. To remove sodium ions, the oligonucleotides were desalted using a 1-k cutoff membrane filter (Microsep Advance Centrifugal Devices with Omega Membrane 1K, PALL). Before adding the oligonucleotides, each filter was washed five times with DEPC water at 15,000 × *g*, 15 °C for 20 min. The desalting step was repeated three times.

#### Characterization.

Analytical RP-HPLC was performed on an Agilent 1200 equipped with a BEH C18 OBD (300 Å, 3.5 µm, 4.6 × 250 mm, 1 mL/min, 60 °C). As solvents 400 mM hexafluoroisopropanol (Fluorochem), 16.3 mM Et_3_N (Merck), pH 8.3 and MeOH (Fluka) were used with a gradient from 5 to 100% MeOH in 39 min. Electrospray ionization-MS spectra were recorded on a Bruker micrOTOF-Q device in negative ionization mode.

### Hippocampal Neurons.

Dissociated rat hippocampal neuron cultures were prepared and maintained as described previously (18). Cells were plated at a density of 30 − 40 × 10^3^ cells/cm^2^ on poly-d-lysine–coated glass-bottom Petri dishes (MatTek). Hippocampal neurons were maintained and matured in a humidified atmosphere at 37 °C and 5% CO_2_ in growth medium (Neurobasal-A supplemented with B27 and GlutaMAX-I; Life Technologies) for 18 to 21 DIV to ensure synapse maturation. All experiments complied with national animal care guidelines and the guidelines issued by the Max Planck Society and were approved by local authorities.

### Transfection of Plasmid DNA.

For transfection of fluorescent proteins and reporters, DIV 17 to 19 neurons were transfected with mCherry-C1 (Clonetech), myr-sfGFP translational reporters (described below) or myr-Venus using Effectene (Qiagen), as previously described (38). pCAG:myr-Venus (64) was a gift from Anna-Katerina Hadjantonakis (Sloan-Kettering Institute, New York) (Addgene plasmid # 32602; n2t.net/addgene:32602; RRID:Addgene_32602). Transfected cells were imaged or fixed (described below) 12 to 18 h posttransfection.

### Transfection of Molecular Beacons.

For transfection of molecular beacons, DIV 17 to 19 neurons were transfected with Attractene (Qiagen). For each MatTek dish, 20 pmol of molecular beacon was resuspended in 75 μL of buffer EC (Qiagen) along with 2 μL of Attractene. The beacon-attractene mixture was incubated for 20 min at room temperature before being added to neurons. Samples were imaged 1 to 12 h posttransfection. We found that imaging beacons <1 or >12 h after transfection resulted in higher background noise, with short incubations resulting in stronger nuclear staining and long incubations resulting in accumulation and aggregation in vesicular structures. Prior to imaging, samples were washed in fresh media to remove nontransfected beacons.

### Electroporation of Plasmid DNA.

Following isolation, 1 million hippocampal neurons were spun down at 500 rpm for 5 min at 4 °C. Cells were resuspended in electroporation solution (Lonza) along with 1 μg pORANGE plasmid DNA construct. Cells were electroporated with the hippocampal/cortical high-viability protocol (Lonza) and resuspended in 2 mL cell growth media. Cells were then plated at a density of 100 × 10^3^ in MatTek dishes coated with poly-d-lysine for 2 h to allow for cell attachment. Following attachment, 1.3 mL media was added, and cells were fed with 500 μL fresh neuronal growth media once a week until the time of experiments.

### Cell Treatments.

Drugs treatments were performed as follows: For puromycin labeling experiments (Puro-PLA), cultured neurons were treated with 10 µM puromycin for 5 to 10 min. For Puro inhibition experiments, cultured neurons were treated with 100 µM puromycin for 5 min. Anisomycin treatment (40 µM) was performed 20 to 45 min prior to puromycin labeling, FRAP or molecular beacon experiments, and was kept in the media through the duration of experiment. mGluR-LTD was induced using DHPG (100 µM) for 5 min and then washed out or in long-term treatments 50 μM for the duration of imaging. cLTP was induced as previously described (38) in E4 buffer supplemented with B27, Glutamax, and MEM amino acids (Thermofisher). The day before the experiment, 50 μM APV (Tocris) was added to neuronal cultures. The day of induction, neurons were incubated in Mg_2_^+^-free E4 media supplemented with 200 μM glycine (Sigma) and 100 μM picrotoxin (Tocris) for 5 min. Following induction, cells were washed and returned to normal media or E4 with calcium and magnesium.

### Imaging of Molecular Beacons.

Investigation of the mRNA dynamics was carried out using a Leica DMi8 total internal reflection fluorescence (TIRF) microscope. Differential interference contrast (DIC) microscopy was used to identify neurons with well-isolated dendrites. mRNA dynamics were recorded for 20 min at a rate of 1 Hz in epi-fluorescence mode. ATTO565 fluorophores were excited using a 561-nm diode laser which provided 1.8 kW/cm^2^ of intensity at the sample plane. ATTO647n was imaged with a 638-nm laser, which produced 2.0 kW/cm^2^ at the sample plane. The fluorescence was recorded with a scientific-CMOS camera (Leica-DFC9000GT). The exposure time was fixed to 200 ms and 2 × 2 camera binning and set the digitalization to 12 bit (low noise) was used to limit the data volume. A 100× oil objective (HC PL APO 100×/1.47 OIL) was used to record a field-of-view of 133 µm × 133 µm. With these settings our pixel size was 130 nm, matching the Nyquist sampling frequency. Neurons were left in their glia-conditioned neurobasal, B27, and Glutamax media because of a Pecon TempController 2000-1 and a Pecon CO_2_-Controller 2000, which kept the samples at 37 °C in a 5% CO_2_ atmosphere.

### Quantification of Beacon Number per Puncta.

To quantify the copy numbers of mRNAs within individual mRNA granules, a commercially synthesized standard containing a single ATTO647N fluorophore anchored on a glass slide (GATTA-Brightness R1 in 0.5 TBE and 11 mM Mg on glass slide; GattaQuant) was used as a normalization standard. This sample was imaged using the same settings for the molecular beacons at different laser powers, ranging from 90 µW (1%) to 6.85 mW (50%), at which point we could observe saturation of the fluorescence. To benchmark the mean counts recorded from a single ATTO647N fluorophore, the maximum intensity around the detected puncta was measured and subtracted the neighboring background. This benchmark intensity was then set to the value *n* = 1 fluorophore and used to normalize the background-subtracted intensity recorded from hippocampal neurons.

### Quantifying mRNA Dynamics.

To extract information on the mRNA dynamics, a custom MATLAB script was used. For each neuron a single dendrite was segmented by manually drawing its profile. The script was divided into filtering the images and rendering the puncta, tracking the mRNAs and extracting information regarding their dynamics. To render the puncta, the background was subtracted by applying a mean filter. The pixel that represents the local maximum around a region of ∼400 nm × 400 nm was then identified and selected. Puncta were rendered in a binary array. This pipeline was repeated each frame of the time series and exported as a movie. mRNA tracking was performed using the Motion-Based Multiple Object Tracking function of MATLAB taking the first 100 frames as training for the model. Any particle that did not appear in consecutive frames was discarded. After tracking was complete, puncta that appear for longer than 20 frames (20 s) were retained and information—such as the puncta coordinates, their distance traveled, their velocities, and directionalities—was extracted. Puncta that moved less than 500 nm throughout the imaging session were classified as fully stationary and were not included in directionality calculations. From the velocity datasets per puncta, the percent of time spent in the confined state was calculated by assessing the total number of frames in which a puncta was detected and how many frames this puncta exhibited a velocity from −500 nm/s to 500 nm/s. Similarly, the percent time spent anterograde or retrograde was calculated by the fraction of time >500 nm/s or <−500 nm/s over the total number of frames detected. For all events detected in the cell, the average time spent in confined, anterograde or retrograde was calculated.

### Translational Inhibitors and mRNA Dynamics Experiments.

Beacons were transfected and imaged as described above, and puromycin and anisomycin were used at the concentrations indicated above. For assessing translational inhibitor effect on mRNA dynamics, two similarly looking neurons (containing a similar number of beacons and a similar morphology) were selected per MatTek dish. The first neuron was imaged as a control reference cell. Following the 20-min imaging window for the control neuron, puromycin was added for 5 min or anisomycin was added for 20 min, prior to the start of imaging for the second, treated, cell. Pairwise assessment (Fig. 3 *B* and *D*) between the control and treated cell per dish was used to assess the effect of the drugs on mRNA dynamic properties.

### Spine Size Experiments.

Neurons DIV 17+ were transfected with myrVenus 12 h prior to imaging and then imaged using a Leica DMi8 TIRF microscope. A 100× oil objective (HC PL APO 100×/1.47 OIL) was used to record a field-of-view of 133 µm × 133 µm using a 488-nm laser line. Samples were imaged for 10 min at baseline one frame every minute. Mock treatment, mGluR-LTD, or cLTP was induced for 5 min, and samples were imaged one frame per minute during the induction phase. Following washout of drugs, neurons were imaged for 90 min postinduction. For anisomycin treatments, anisomycin was added 20 min prior to the start of the experiment and kept in the media continuously throughout the experiment. Drift was corrected using the built-in correct three-dimensional drift plugin in ImageJ/FIJI. An area of 5 to 10 spines per dendrite were then quantified over the imaging window, using the mean size of the first 10 baseline frames for normalization.

### Plasticity and mRNA Dynamics Experiments.

Beacons were transfected and imaged and plasticity was induced as described above. For assessing the effect of plasticity on mRNA dynamics, two similar neurons (beacon number and morphology) were selected per MatTek dish. The first neuron was imaged as a control reference neuron. Following the 20-min imaging window for the control neuron, plasticity was induced and the stimulation washed out, prior to commencing imaging of the second, treated, cell. Pairwise assessment (Fig. 4 *A* and *C*) between the control and treated cell per dish was used to assess the effect of plasticity on mRNA dynamic properties.

### FISH.

Hippocampal neurons (DIV 18+) expressing mCherry were fixed in 4% paraformaldehyde lysine phosphate buffer pH 7.4 supplemented with 2.5% sucrose for 15 to 20 min. Cells were permeabilized for 10 min in PBS containing 0.5% Triton-X 100 (Sigma). Target specific in situ hybridization was performed using StellarisTM probes (LGC Bioresearch) as previously described (10). Following fixation, cells were washed in PBS + 5 mM MgCl_2_, followed by dehydration in 80% ethanol overnight at −20 °C. Following rehydration samples were washed 2× in 1× sodium citrate (SSC) buffer, followed by a 5-min wash in 2× SSC + 30% formamide for 5 min at 37 °C. Biotin-labeled probes for *Camk2a*, *β-actin*, and *Psd95* (Stellaris, Biosearch Technology) were diluted into 100 μL hybridization buffer and incubated with cells for 4 h at 37 °C. Following probe hybridization, samples were washed twice in 2× SSC + 30% formamide for 30 min each, followed by five 1× SSC washes. After completion of in situ hybridization, samples were washed with phosphate buffered saline (PBS) and subsequently processed for immunofluorescence. Immunofluorescence was performed on fixed and permeabilized samples with or without in situ hybridization using the following protocol: samples were incubated in biotin-free blocking buffer (4% biotin free BSA in PBS) for 30 min and then incubated for 1.5 h at room temperature or overnight at 4 °C with primary antibodies in blocking buffer. After three washes in PBS for 5 min each, samples were incubated in blocking buffer (4% goat serum in PBS) for 1 to 2 h with secondary antibodies. The following antibodies were used: rabbit anti-biotin (Cell Signaling; 1:1,000), rat anti-mCherry (Abcam; 1:1,000), goat anti-rabbit Alexa 488, and goat anti-rat Alexa 568. Samples were imaged using Zeiss LSM780/880 confocal microscopes and a 63× oil objective. Images spanning the entire volume of a neuron were obtained and analyzed using ImageJ. The distance to the nearest spine was measured by performing line-scan analysis through a punctum of interest to the base of nearby spines. From the value of peak intensity of the mRNA puncta (the centroid) we assessed the recorded the shortest distance as the RNA to spine distance.

For FISH experiments in 1D&E, a similar procedure was performed using Quasar 570 labeled smFISH probes (Stellaris). Forty-four probes per transcript were used. Following wash steps samples were imaged with a scientific-CMOS camera (Leica-DFC9000GT). The exposure time was fixed to 700 ms. A 100× oil objective (HC PL APO 100×/1.47 OIL) was used to record a field-of-view of 133 µm × 133 µm. On dendritic segments, particles were automatically detected based on size (Fiji) and the integrated density of the particles were recorded from 50 mRNA puncta per dendritic segment.

### FRAP Translational Reporters.

A codon-optimized superfolder GFP was custom synthesized (Eurofins) and cloned into a plasmid backbone driven by a CMV promoter. The 3′UTRs corresponding to the most highly dendritically localized isoforms (41) for *Camk2a*, *β-actin*, and *Psd95* were cloned upstream of a SV40 polyadenylation sequence. sfGFP reporters were transfected into neurons 12 h prior to imaging. Cells were imaged at 63× on a LSM780 (NA 1.4, PSF: 0.240/0.258/0.729), with a temperature-regulated environmental chamber. Cells were maintained in E4 buffer (38) supplemented with B27, Glutamax, and 1× MEM amino acids (Thermofisher). Whole-cell photobleaching was accomplished using a 488-nm argon laser (1.49 mW) with an intensity of 2,900 kW/cm^2^ for 40 to 50 s. Cells were imaged at 0.067 Hz for 2 min prior to and 10 min following the bleaching step. Fluorescence intensity was measured in a 50-μm dendritic segment from the raw image. FRAP was calculated from background-corrected fluorescence intensity by normalizing the change in fluorescence (*F*–*F*_0_) to prephotobleaching fluorescence (*F*_i_). Mobile fraction and *t*_1/2_ values were extracted from data fitted to a one-phase exponential association.

### Endogenous FRAP.

Venus tagging pORANGE CRISPR/Cas9 constructs (42) were generated from previously described GFP tagging plasmids (Addgene plasmids #131477, #131479, #131484; gifts from Harold MacGillavry, Universiteit Utrecht, Utrecht, Netherlands). Neurons were electroporated (see above) at the day of plating and maintained until DIV 17 to 21 for FRAP experiments. FRAP and imaging was carried out using a Leica DMi8 TIRF microscope. FRAP was performed using a 488-nm laser, providing 7.78-mW/cm^2^ intensity. Cells were imaged for 2 min at baseline with a 488-nm LED every 15 s prior to bleaching for a baseline measurement. Whole-cell bleaching was performed with 20 to 30 s of bleaching (PSD-95) or 50 to 70 s of bleaching (CAMK2a and β-ACTIN). Cells were the imaged in epi-fluorescence mode with the LED every 15 s for 60 min. Fluorescence intensity was measured in a 50-μm dendritic segment from the raw image. FRAP was calculated from background-corrected fluorescence intensity by normalizing the change in fluorescence (*F*–*F*_0_) to prephotobleaching fluorescence (*F*_i_). Mobile fraction and *t*_1/2_ values were extracted from data fitted to a one phase exponential association.

### Puro-PLA.

Detection of newly synthesized proteins by PLA was performed as previously described (5, 10, 45). Immunostaining using mouse antipuromycin (Kerafast; 1:500) antibody in combination with rabbit anti–β-actin (Abcam; 1:1,000), rabbit anti–PSD-95 (cell signaling technologies; 1:1,000), or rabbit anti-Camk2a (Thermo; 1:1,000) was performed overnight at 4 °C. Following 5× PBS washes, PLA was performed (Sigma). Rabbit PLA^plus^ and mouse PLA^minus^ probes were used. PLA was performed according to the manufacture’s guidelines. Following PLA, anti-Map2 immunostaining (guinea pig anti-Map2, Cell Signaling; 1:5,000) was performed to label dendrites. Samples were imaged using a 40× oil objective on a LSM780 or LSM880. *Z*-stacks (0.43 μm) spanned the entire volume of imaged neurons. Images were analyzed using ImageJ. A 100-μm segment of the dendrite was assessed for the number of Puro-PLA puncta and the density of signal was calculated.

### Statistics.

Statistical significance, the tests performed, and the number of cells/replicates are indicated in the figure legends. Statistical analysis was performed using GraphPad Prism.

## Supplementary Material

Supplementary File

Supplementary File

Supplementary File

Supplementary File

Supplementary File

Supplementary File

Supplementary File

## Data Availability

Analysis scripts are deposited on GitHub (https://gitlab.mpcdf.mpg.de/mpibrcpo/beacon-tracker). Plasmids will be made available through Addgene. All other data, including sequences used for the molecular beacons, are available in the article and *SI Appendix*.
