# Supplementary material for: Differential regulation of local mRNA dynamics and translation following long-term potentiation and depression
Source: Proc Natl Acad Sci U S A. 2021 Mar 26;118(13):e2017578118. doi: 10.1073/pnas.2017578118 (PMC8020670; doi:10.1073/pnas.2017578118)
Supplement: Supplementary File [file pnas.2017578118.sapp.pdf]

PNAS
www.pnas.org

Supplementary Information for

Differential regulation of local mRNA dynamics and translation following long-term potentiation and depression.

Paul G Donlin-Asp[1], Claudio Polisseni[1], Robin Klimek[2], Alexander Heckel[2], Erin M Schuman[1*]
1: Max Planck Institute for Brain Research, Frankfurt Germany
2: Goethe-University Frankfurt, Institute for Organic Chemistry and Chemical Biology, Frankfurt, Germany

*To whom correspondence should be addressed

Email: erin.schuman@brain.mpg.de

This PDF file includes:

Figures S1 to S8
Tables S1 to S2
Legends for Movies S1 to S6

Other supplementary materials for this manuscript include the following:

Movies S1 to S6

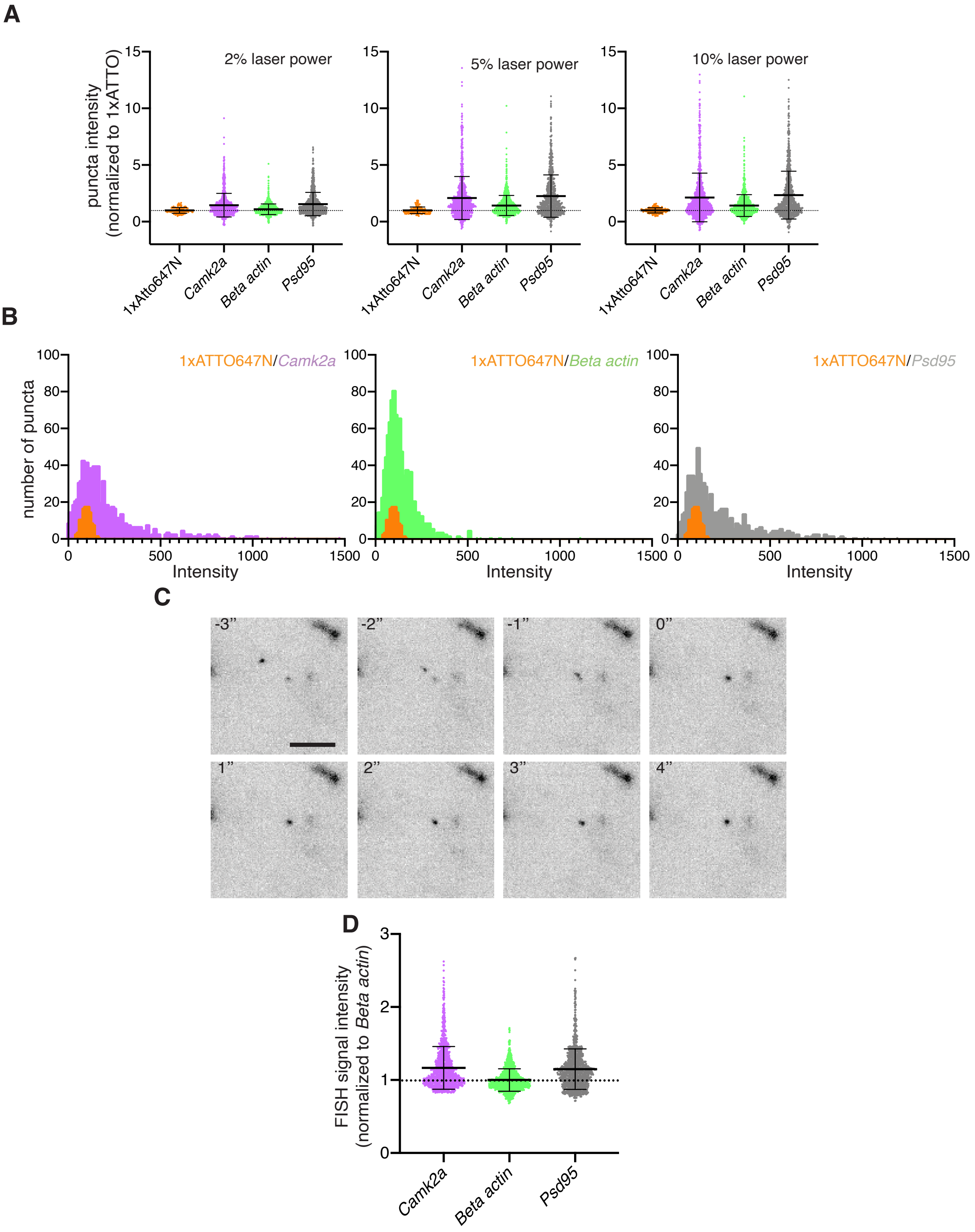
A
puncta intensity
(normalized to 1xATTO)
2% laser power
5% laser power
10% laser power
1xAtto647N
Camk2a
Beta actin
Psd95
B
number of puncta
1xATTO647N/Camk2a
1xATTO647N/Beta actin
1xATTO647N/Psd95
Intensity
C
-3''
-2''
-1''
0''
1''
2''
3''
4''
D
FISH signal intensity
(normalized to Beta actin)
Camk2a
Beta actin
Psd95

Supplemental Figure 1: Characterization of mRNA organizational states.

A. Quantification of molecular beacon intensity in dendrites showed a heterogenous distribution pattern of copy numbers of mRNAs per granule in transcript specific manner. 1x Atto647N (3/150); *Camk2a* (19/921), *Beta actin* (21/1003), *Psd95* (18/829) (cells/number of individual mRNA granules analyzed). Data shown acquired at 2, 5%, and 10% laser power, showing that the pattern of distribution holds true at various laser intensities. **B.** Plots of raw intensities of beacons and the Gatta quant standard highlighted the existence of single mRNA transport states along with higher order complex states. Data shown acquired at 10% laser intensity. **C.** Stills from a *Camk2a* 647 beacon movie (supplemental video 5) where a RNA-RNA fusion event (0”) is seen. Scale bar = 5um. **D.** Quantification of smFISH within dendrites showed a heterogenous distribution pattern of copy numbers of mRNAs per granule in transcript specific manner. *Camk2a* (15/1005), *Beta actin* (15/1005), *Psd95* (15/1005) (cells/number of individual mRNA granules analyzed).

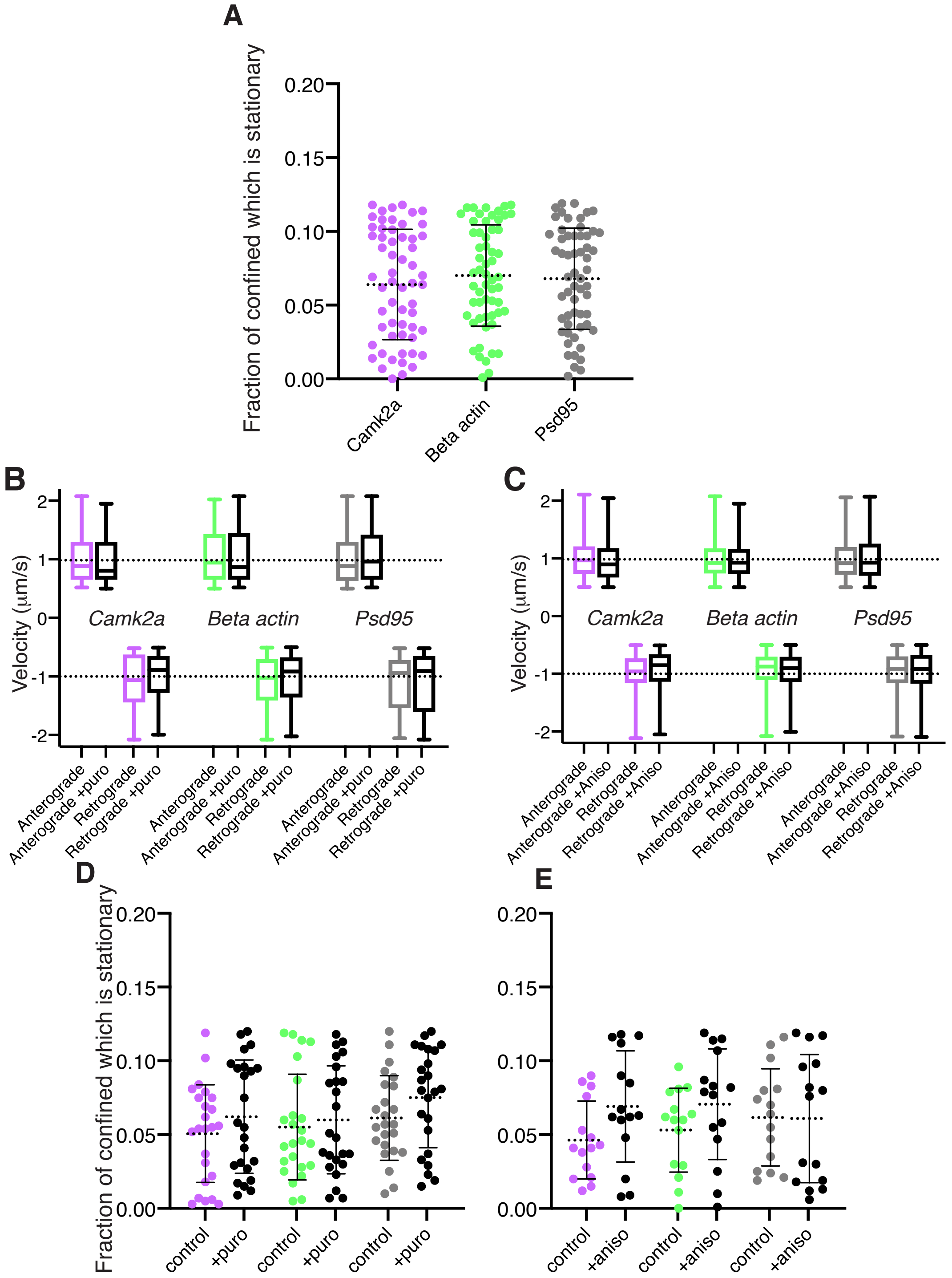

A
Fraction of confined which is stationary
0.20
0.15
0.10
0.05
0.00
Camk2a
Beta actin
Psd95
B
Velocity (µm/s)
2
1
0
-1
-2
Camk2a
Beta actin
Psd95
Anterograde
Anterograde +puro
Retrograde
Retrograde +puro
C
Velocity (µm/s)
Camk2a
Beta actin
Psd95
Anterograde
Anterograde +Aniso
Retrograde
Retrograde +Aniso
D
Fraction of confined which is stationary
control
+puro
E
control
+aniso

Supplemental Figure 2: Stationary vs confined analysis for baseline and translational inhibitor experiments

A. Quantification of the confined population of Figure 2C which is stationary. Mean +/- SD: Camk2a 0.0640 +/- 0.0374; Beta actin 0.0701 +/- 0.0343; Psd95 0.0680 +/- 0.0344. **B.** Quantification of mRNA velocity for anterograde and retrograde control vs puro treated samples for *Camk2a (1.015 +/- 0.442 vs 0.987 +/- 0.449; -1.079 +/- .478 vs -0.991 +/- 0.420)* , *Beta actin (1.052+/- 0.489 vs 1.030 +/- 0.475; -1.130 +/- 0.449 vs -1.040 +/- 0.430)* and *Psd95 (0.988 +/- 0.414 vs 1.065 +/- 0.457; -1.111 +/- 0.484 vs -1.119 +/- .510)*. n = 29 cells per condition. **C.** Quantification of mRNA velocity for anterograde and retrograde control vs anis treated samples for *Camk2a (1.011 +/- 0.351 vs 0.9543 +/- 0.338; -0.990 +/- .335 vs -0.931 +/- 0.332)* , *Beta actin (0.993 +/- 0.334 vs 0.973 +/- 0.317; -0.942 +/- 0.308 vs -0.958 +/- 0.317)* and *Psd95 (0.990 +/- 0.343 vs 1.004 +/- 0.378; -0.973 +/- 0.340 vs -0.971 +/- .371)*. n = 15 cells per condition. **D.** Quantification of the confined population of Figure 3B which is stationary. Mean +/- SD: Camk2a 0.0507 +/- 0.0331 vs. 0.0622 +/- 0.0384; Beta actin 0.0541 +/- 0.0358 vs. 0.0599 +/- 0.0366; Psd95 0.0613 +/- 0.0287 vs. 0.0751 +- 0.03402. **E.** Quantification of the confined population of Figure 3D which is stationary. Mean +/- SD: Camk2a 0.0463 +/- 0.0264 vs. 0.0691 +/- 0.0377; Beta actin 0.0530 +/- 0.0285 vs. 0.0706 +/- 0.0376; Psd95 0.0616 +/- 0.0329 vs. 0.0609 +- 0.0433.

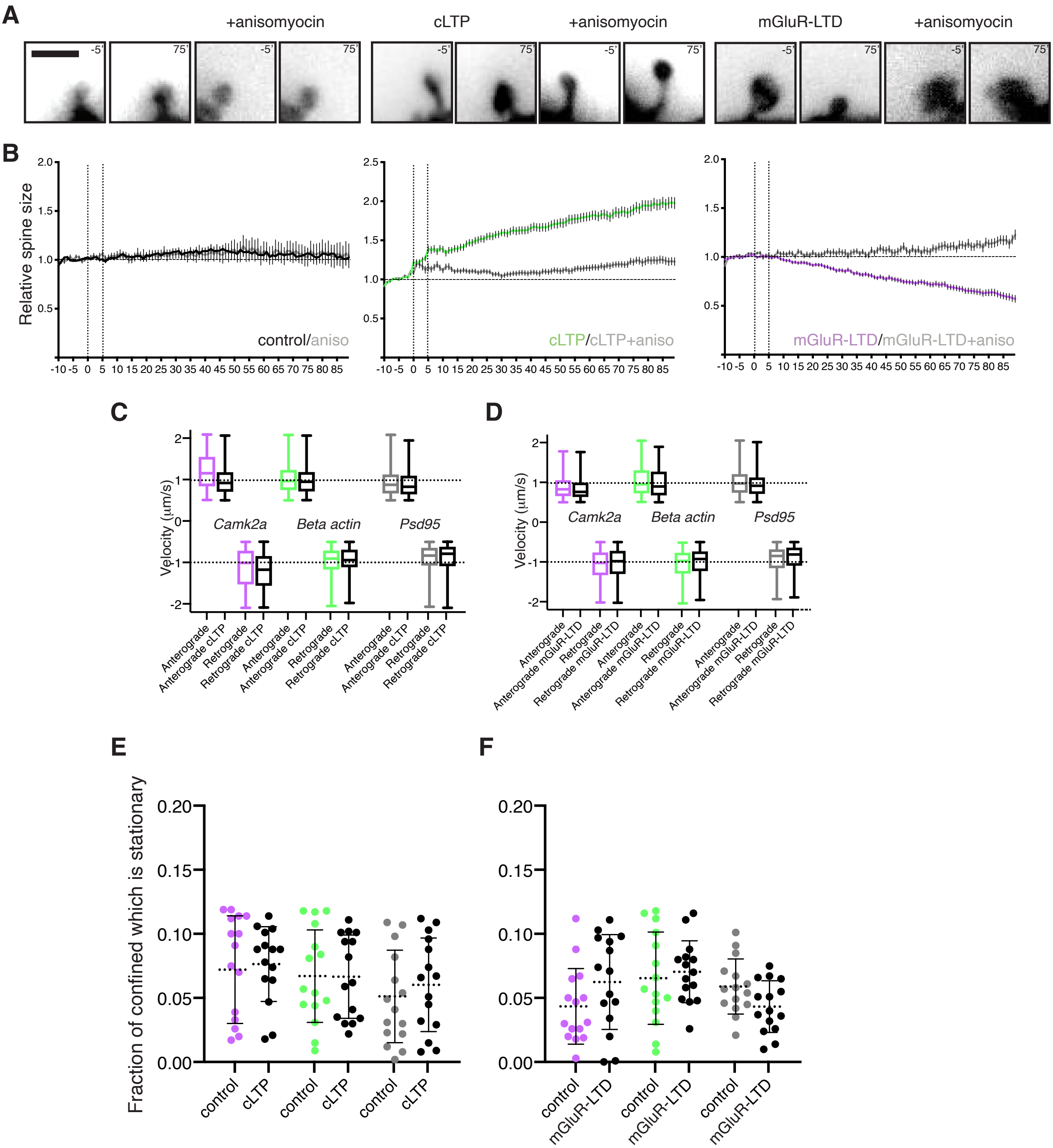
A
+anisomyocin
cLTP
+anisomyocin
mGluR-LTD
+anisomyocin
-5'
75'
B
Relative spine size
control/aniso
cLTP/cLTP+aniso
mGluR-LTD/mGluR-LTD+aniso
-10 -5 0 5 10 15 20 25 30 35 40 45 50 55 60 65 70 75 80 85
C
Velocity (μm/s)
Camk2a
Beta actin
Psd95
Anterograde
Anterograde cLTP
Retrograde
Retrograde cLTP
D
Velocity (μm/s)
Camk2a
Beta actin
Psd95
Anterograde
Anterograde mGluR-LTD
Retrograde
Retrograde mGluR-LTD
E
Fraction of confined which is stationary
control
cLTP
F
control
mGluR-LTD

Supplemental Figure 3: Structural plasticity changes with dendritic spines during cLTP and mGluR-LTD is protein synthesis-dependent

A. Example images of spines before (-5’) and after (75’) mock or plasticity stimulation in the absence or presence of anisomycin. Scale bar = 1um. **B.** Quantification of spine size over time demonstrated that cLTP and mGluR-LTD require protein synthesis for their manifestation, as spine enlargement or shrinkage is blocked by pretreatment with anisomycin. -10’-0 minutes = baseline measurement, 0-5’ = stimulation phase, 5-90’ minutes = post plasticity induction. 5-10 spines per cell were analyzed, with 20 cells in total assessed per condition. **C.** Quantification of mRNA velocity for anterograde and retrograde for *Camk2a*, *Beta actin* and *Psd95* for control vs cLTP induced samples. n = 14 cells per condition. **D.** Quantification of mRNA velocity for anterograde and retrograde for *Camk2a*, *Beta actin* and *Psd95* for control vs mGluR-LTD induced samples. n =14 cells per condition. **E.** cLTP induction did not change the overall fraction of the mRNA population which was stationary. **F.** mGluR-LTD induction did not change the overall fraction of the mRNA population which was stationary.

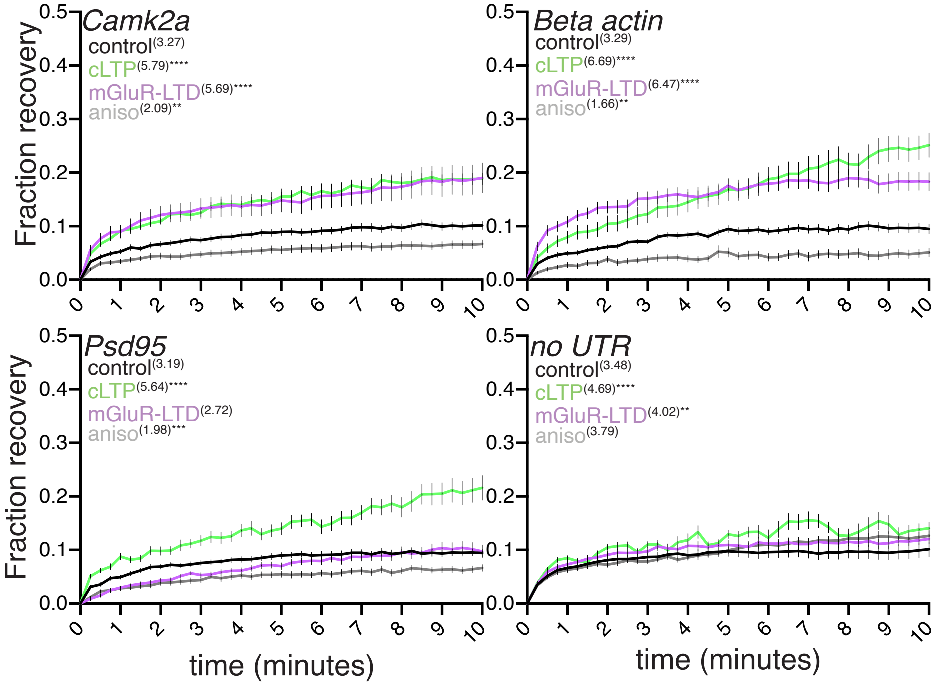

Camk2a
control(3.27)
cLTP(5.79)****
mGluR-LTD(5.69)****
aniso(2.09)**
Beta actin
control(3.29)
cLTP(6.69)****
mGluR-LTD(6.47)****
aniso(1.66)**
Psd95
control(3.19)
cLTP(5.64)****
mGluR-LTD(2.72)
aniso(1.98)***
no UTR
control(3.48)
cLTP(4.69)****
mGluR-LTD(4.02)**
aniso(3.79)
Fraction recovery
time (minutes)

Supplemental Figure 4: FRAP recovery curves for translational reporters

Fluorescence recovery curves for all constructs under all conditions. **/***/**** p <.01/.001/.0001. Dunn's multiple comparisons test for treated vs control condition for each construct; n = >14 cells per condition. Values in parentheses area under the curve for each condition. (area under the curve- AUC: $AUC_{cLTP}/AUC_{control}$ CAMK2a: 1.771; $AUC_{mGluR\text{-}LTD}/AUC_{control}$ CAMK2α: 1.740, $AUC_{cLTP}/AUC_{control}$ BETA ACTIN: 2.033; $AUC_{mGluR\text{-}LTD}/AUC_{control}$ BETA ACTIN: 1.967, $AUC_{cLTP}/AUC_{control}$ PSD-95: 1.768; $AUC_{mGluR\text{-}LTD}/AUC_{control}$ PSD-95: 0.852, $AUC_{aniso}/AUC_{control}$ no UTR: 1.089; CAMK2α: 0.639; BETA ACTIN: 0.504; PSD-95: 0.620)

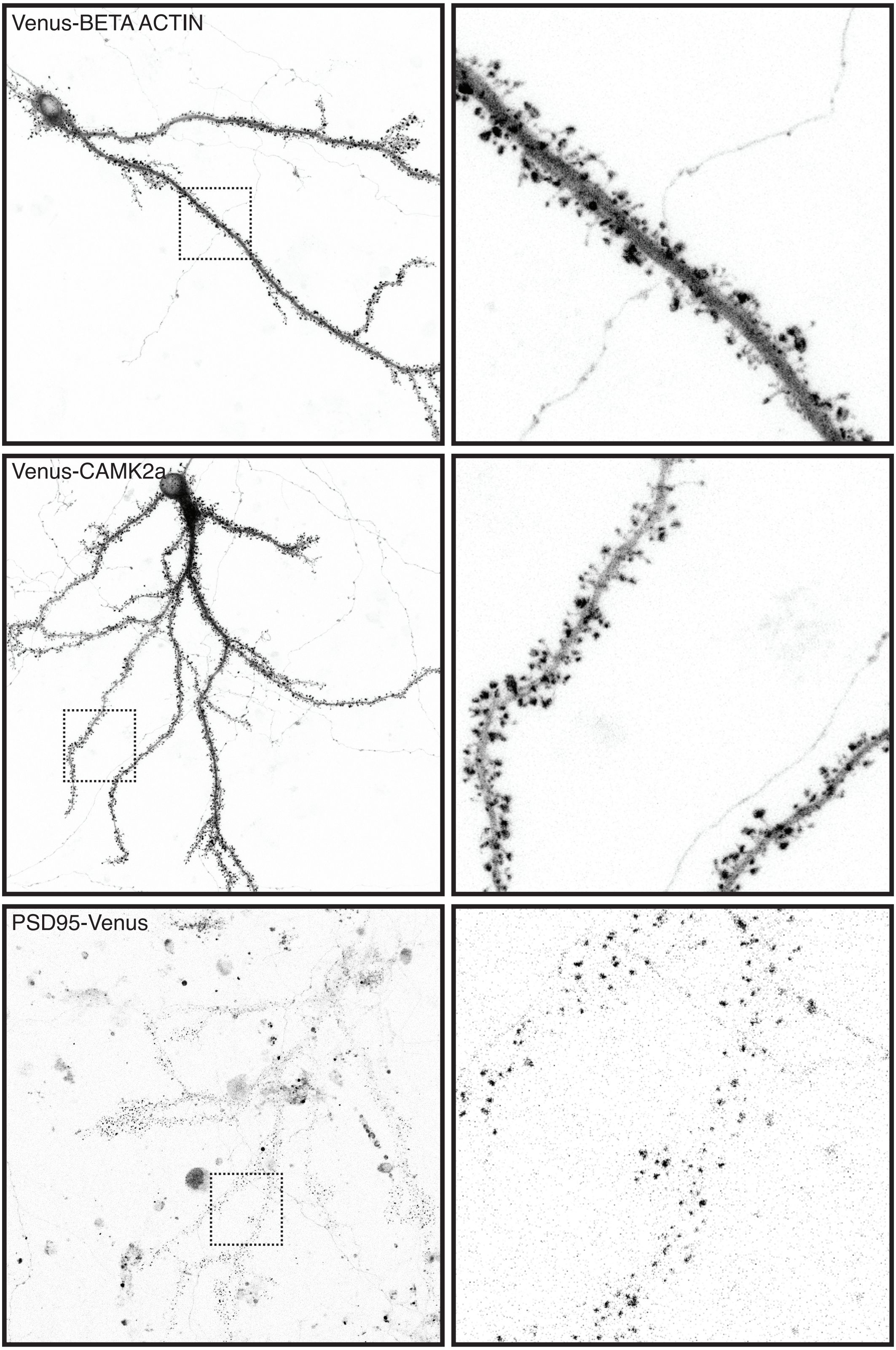
Venus-BETA ACTIN
Venus-CAMK2a
PSD95-Venus

Supplemental Figure 5: CRISPR/Cas9 labeled examples for Figure 5

A. Example of a Venus-BETA ACTIN labeled neuron, DIV21. Inset 40um x 40um. **B.** Example of a Venus-CAMK2a labeled neuron, DIV21. Inset 40um x 40um. **C.** Example of a PSD-95-Venus labeled neuron, DIV21. Inset 40um x 40um.

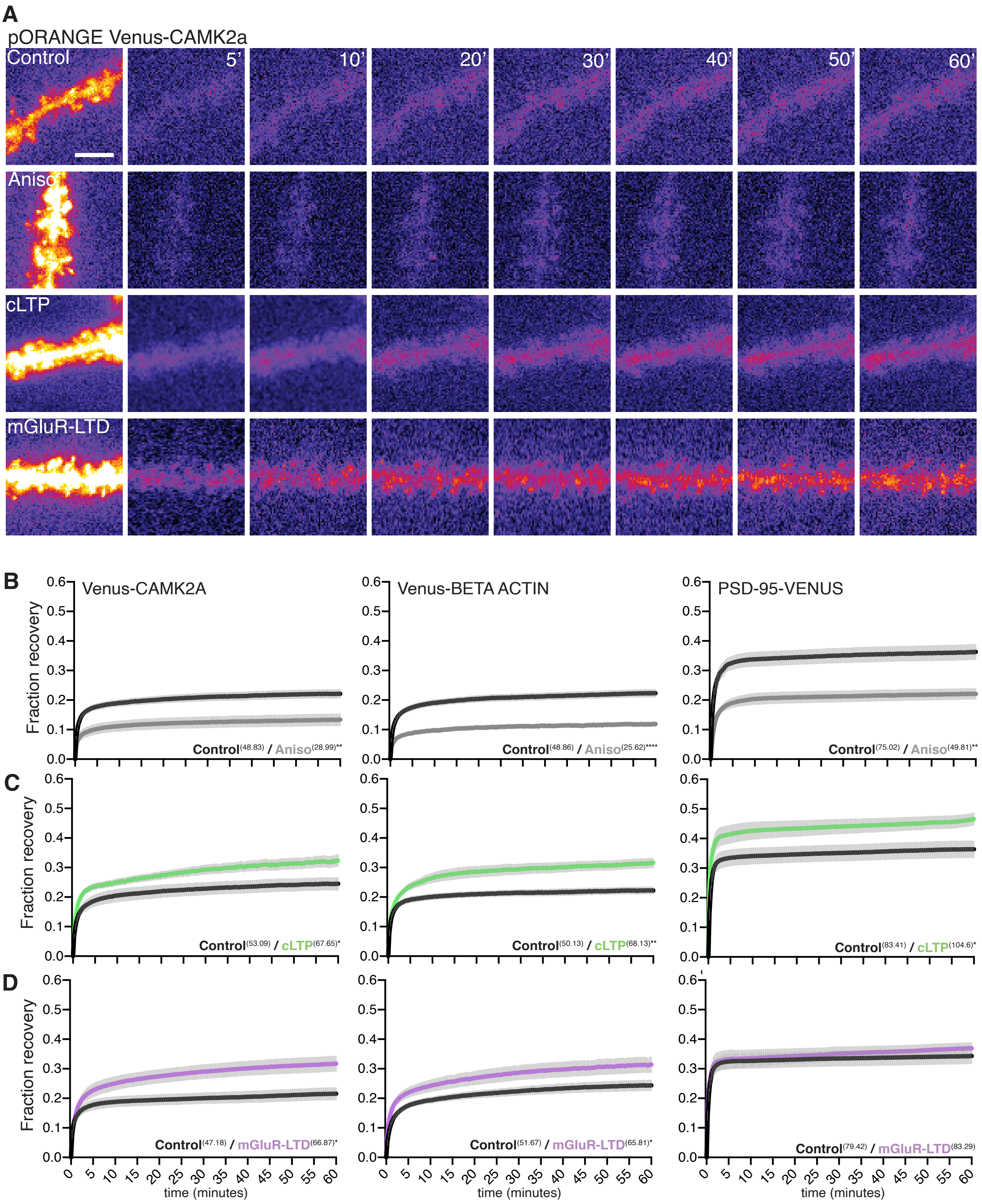

A
pORANGE Venus-CAMK2a
Control
5'
10'
20'
30'
40'
50'
60'
Aniso
cLTP
mGluR-LTD
B
Venus-CAMK2A
Venus-BETA ACTIN
PSD-95-VENUS
Fraction recovery
Control(48.83) / Aniso(28.99)**
Control(48.86) / Aniso(25.62)****
Control(75.02) / Aniso(49.81)**
C
Control(53.09) / cLTP(67.65)*
Control(50.13) / cLTP(68.13)**
Control(83.41) / cLTP(104.6)*
D
Control(47.18) / mGluR-LTD(66.87)*
Control(51.67) / mGluR-LTD(65.81)*
Control(79.42) / mGluR-LTD(83.29)
time (minutes)

Supplemental Figure 6: FRAP examples for Figure 5

A. FRAP recovery examples for Venus-CAMK2a during Control, Aniso, cLTP and mGluR-LTD. Scale bar = 5um. **B.** Fluorescence recovery curves for Venus-CAMK2a, Venus-BETA ACTIN and PSD-95-Venus with and without anisomycin treatment. **/**** p <.01/.0001. Dunn's multiple comparisons test. n = 10 cells per condition. Values in parentheses are the area under the curve for each condition. ($AUC_{aniso}/AUC_{control}$ CAMK2α: 0.594; BETA ACTIN: 0.524; PSD-95: 0.664). **C.** Fluorescence recovery curves for Venus-CAMK2a, Venus-BETA ACTIN and PSD-95-Venus with and without cLTP induction. */** p <.05/.01. Dunn's multiple comparisons test. n = 10 cells per condition. Values in parentheses area under the curve for each condition. ($AUC_{cLTP}/AUC_{control}$ CAMK2α: 1.274; BETA ACTIN: 1.360; PSD-95: 1.254). **D.** Fluorescence recovery curves for Venus-CAMK2a, Venus-BETA ACTIN and PSD-95-Venus with and without mGluR-LTD induction. * p <.05. Dunn's multiple comparisons test. n=9-10 cells per condition. Values in parentheses are the area under the curve for each condition. ($AUC_{cLTP}/AUC_{control}$ CAMK2α: 1.417; BETA ACTIN: 1.279; PSD-95: 1.049)

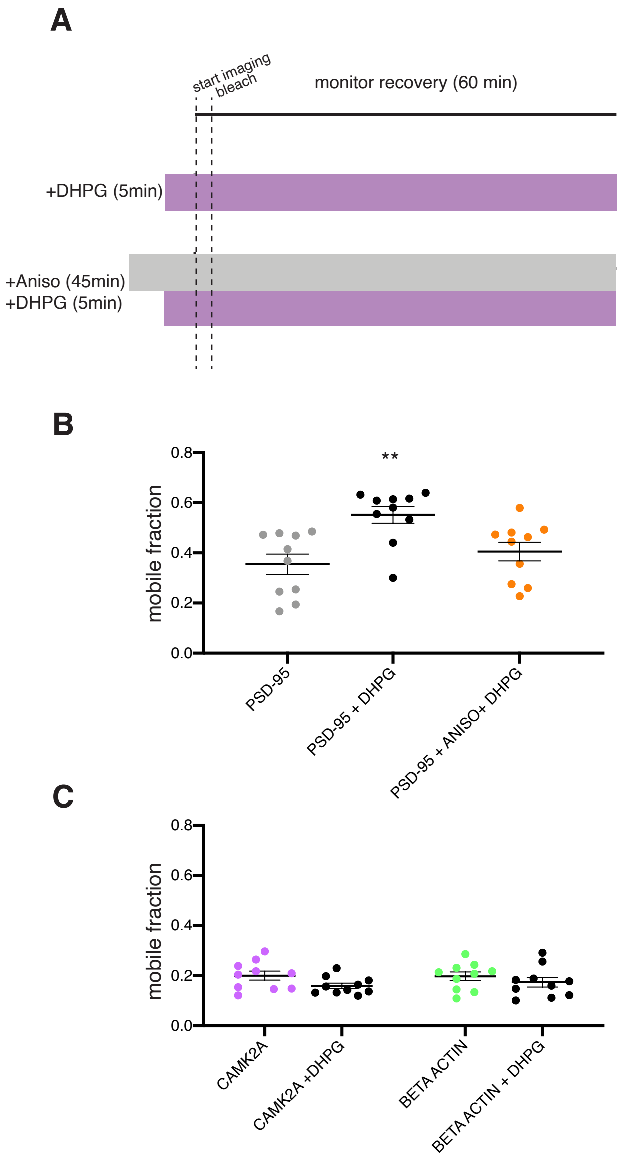
A
start imaging
bleach
monitor recovery (60 min)
+DHPG (5min)
+Aniso (45min)
+DHPG (5min)
B
mobile fraction
0.8
0.6
0.4
0.2
0.0
**
PSD-95
PSD-95 + DHPG
PSD-95 + ANISO+ DHPG
C
mobile fraction
0.8
0.6
0.4
0.2
0.0
CAMK2A
CAMK2A +DHPG
BETA ACTIN
BETA ACTIN + DHPG

Supplemental Figure 7: Long-term DHPG stimulation induces PSD-95 protein synthesis

A. Scheme of workflow for the treatment and visualization of long-term DHPG stimulation. Cells were pretreated with DHPG for 5 minutes, then imaged 2 minutes every 15 seconds to acquire a baseline measurement. The cells were then bleached- and the fluorescence recovery was then monitored every 15 seconds for 60 minutes. For controls, either no treatment or treatment with the protein synthesis inhibitor anisomycin prior to addition of DHPG was used for comparison. **B.** Mobile population during the FRAP recovery for PSD-95-Venus during control, DHPG and anisomycin +DHPG treatment. n = 10 cells per condition. Two-tailed paired T-test. ** $p<.01$. **C.** Mobile population during the FRAP recovery for Venus-CAMK2a and Venus-BETA ACTIN during control and DHPG treatment. n = 10 cells per condition.

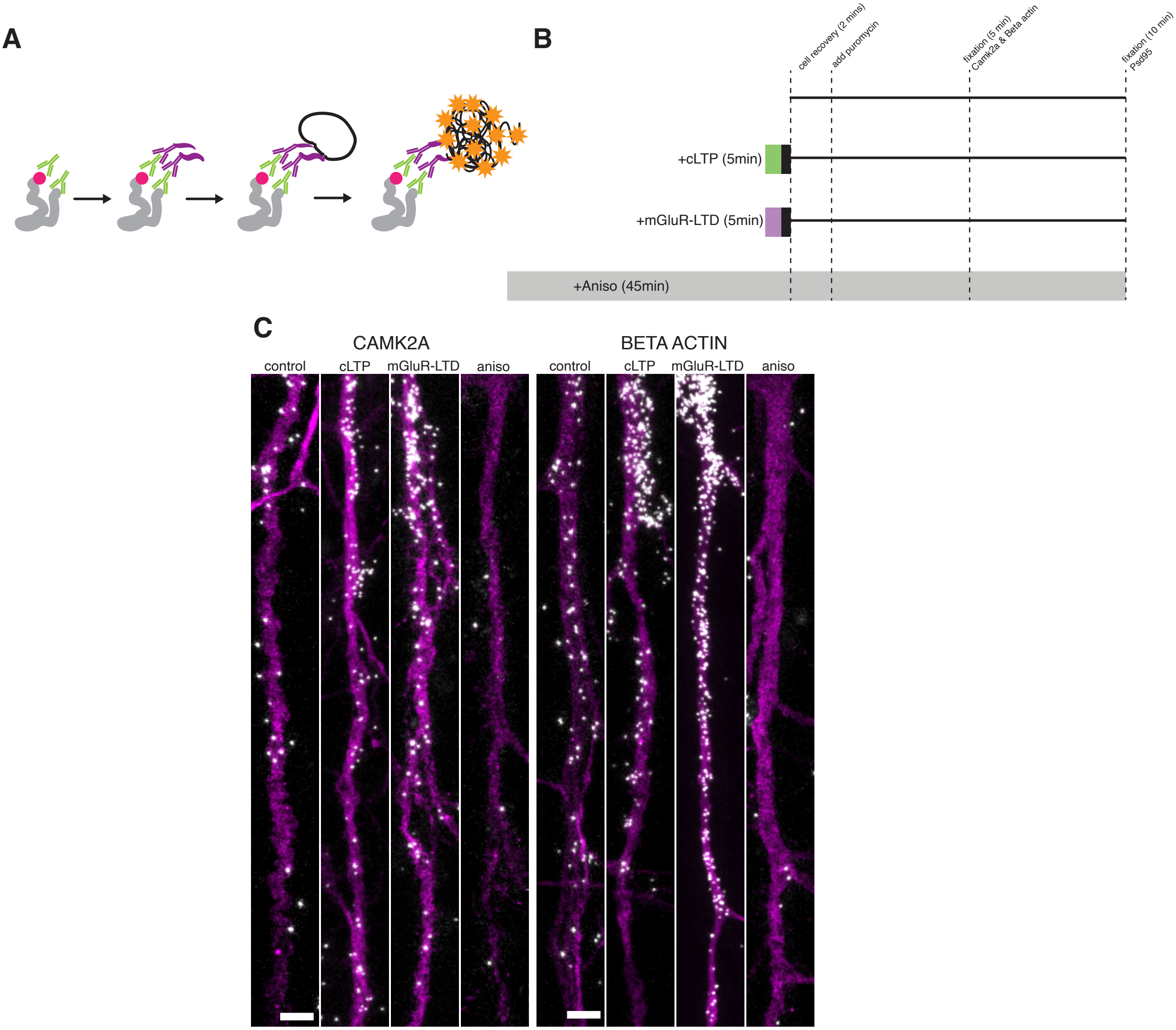
A
B
cell recovery (2 mins)
add puromycin
fixation (5 min)
Camk2a & Beta actin
fixation (10 min)
Psd95
+cLTP (5min)
+mGluR-LTD (5min)
+Aniso (45min)
C
CAMK2A
control
cLTP
mGluR-LTD
aniso
BETA ACTIN
control
cLTP
mGluR-LTD
aniso

Supplemental Figure 8: Puro-PLA examples for Figure 6

A. Schematic representation of the Puro-PLA method (see methods). Nascent peptides (gray) are labeled metabolically with puromycin (pink) for 5-10 minutes. A protein of interest and the puromycin tag are detected by primary antibodies (green) and then detected by PLA oligo modified secondaries (purple). With a rolling chain amplification (orange) newly synthesized proteins can be visualized *in situ*. **B.** Workflow for detecting newly synthesized CAMK2a, BETA ACTIN and PSD-95. Treatments were applied and washed out (aside from anisomycin which is maintained throughout the workflow) and cells were allowed to recover for 2 minutes prior to the addition of puromycin (10uM). CAMK2a and BETA ACTIN were fixed after 5 minutes of puromycin incubation, and PSD-95 was fixed after 10 minutes. **C.** Example images for (Figure 6G) for CAMK2a and BETA ACTIN Puro-PLA signal (gray) within dendrites (MAP2, magenta). Scale bar = 5um.

Supplemental Table 1: FRAP statistics for Figure 5

	Motile fraction (mean +/- SD	t1/2 (s)
**BETA ACTIN**	.1157 +/- .04728	74.64 +/- 49.062
**BETA ACTIN+aniso**	.04919 +/- .03067	75.54 +/- 51.15
**BETA ACTIN + cLTP**	.3565 +/- .1376	114 +/- 46.938
**BETA ACTIN + mGluR-LTD**	.1840 +/- .08830	60 +/- 38.322
**CAMK2**	.09946 +/- .03966	82.50 +/- 55.026
**CAMK2 +aniso**	.05238 +/- .02454	75.96 +/- 33.739
**CAMK2 + cLTP**	.1845 +/- .05424	91.50 +/- 40.692
**CAMK2 + mGluR-LTD**	.1796 +/- .1539	92.34 +/- 213.60
**PSD-95**	.09471 +/- .03452	63.84 +/- 31.14
**PSD-95 + aniso**	.06705 +/- .02575	123.54 +/- 113.16
**PSD-95 + cLTP**	.2147 +/- .1540	124.92 +/- 135.84
**PSD-95 + mGluR-LTD**	.1023 +/- .07338	145.62 +/- 112.68
**No UTR**	.1130 +/- .06654	61.44 +/- 70.38
**No UTR + aniso**	.1506 +/- .09210	145.80 +/- 195.50
**No UTR + cLTP**	.1473 +/- .07884	67.98 +/- 56.418
**No UTR + mGluR-LTD**	.1167 +/- .04943	54.822 +/- 38.55

Supplemental Table 2: FRAP statistics for Figure 6

	Motile fraction	t1/2 (s)
**BETA ACTIN**	.2103 +/- .01248	83.82 +/- 46.60
**BETA ACTIN+aniso**	.1129 +/- .007263	119.10 +/- 84.06
**CAMK2a**	.2095 +/- .01399	73.62 +/- 62.64
**CAMK2a + aniso**	.1260 +/- .01946	114.48 +/- 117.66
**PSD-95**	.3499 +/- .02560	41.74 +/- 24.73
**PSD-95 + aniso**	.2131 +/- .01961	62.52 +/- 35.99
**BETA ACTIN**	.2116 +/- 0.0323	51.01 +/- 28.22
**BETA ACTIN+cLTP**	.295 +/- 0.0571	98.04 +/- 33.38
**CAMK2a**	.232 +/- .06864	129.78 +/- 100.8
**CAMK2a + cLTP**	.2995 +/- 0.04688	117.6 +/- 91,5
**PSD-95**	.3521 +/- 0.08919	32.95 +/- 30.61
**PSD-95 + cLTP**	.4456 +/- .07097	64.80 +/- 143.38
**BETA ACTIN**	.2282 +/- .04881	142.20 +/- 94.14
**BETA ACTIN+mGluR-LTD**	.3168 +/- .07219	130.38 +/- 57.44
**CAMK2a**	.2005 +/- 0.05813	53.49 +/- 32.45

**CAMK2a + mGluR-LTD**	.2935 +/- .07424	136.68 +/- 70.92
**PSD-95**	.3342 +/- .08421	62.46 +/- 33.804
**PSD-95 + mGluR-LTD**	.3667 +/- .06901	43.61 +/- 29.33

Supplemental Table 3: FRAP statistics for Supplemental Figure 7

	Motile fraction	t1/2 (s)
**PSD-95**	.3550 +/- .1276	72.84 +/- 33.97
**PSD-95 + DHPG**	.5521 +/- .1067	73.92 +/- 21.31
**PSD-95 + Aniso + DHPG**	.4054 +/- .1180	70.14 +/- 30.50
**BETA ACTIN**	.1977 +/- .05417	73.08 +/- 40.17
**BETA ACTIN+DHPG**	.1743 +/- .06116	60.42 +/- 26.84
**CAMK2a**	.2003 +/- .05707	125.52 +/- 79.10
**CAMK2a + DHPG**	.1596 +/- .03449	135.72 +/- 48.91

Supplemental video 1: Camk2a 647N beacon in live hippocampal neurons

Supplemental video 2: Beta actin 647N beacon in live hippocampal neurons

Supplemental video 3: Psd95 647 N beacon in live hippocampal neurons

Supplemental video 4: GFP 565 beacon in live hippocampal neurons

Supplemental video 5: *Camk2a*:*Camk2a* fusion event detected in dendrites

Supplemental video 6: Beta actin example from Figure 2A&B
